# Two-dimensional Health State Map to define metabolic health using separated static and dynamic homeostasis features: a proof-of-concept study

**DOI:** 10.1093/nsr/nwae425

**Published:** 2024-11-26

**Authors:** Yanpu Wu, Xinyan Zhang, Liang Sun, Qingqing Wu, Xiaoping Liu, Yueyi Deng, Zhenzhen Lu, Zhongxia Li, Chaoming Deng, Ruikun He, Luyun Zhang, Rong Zeng, Xuguang Zhang, Luonan Chen, Xu Lin

**Affiliations:** Shanghai Institute of Nutrition and Health, Chinese Academy of Sciences, Shanghai 200031, China; BYHEALTH Institute of Nutrition & Health, Guangzhou 510799, China; Key Laboratory of Systems Health Science of Zhejiang Province, School of Life Science, Hangzhou Institute for Advanced Study, University of Chinese Academy of Sciences, Hangzhou 310024, China; Key Laboratory of Systems Biology, Center for Excellence in Molecular Cell Science, Shanghai Institute of Biochemistry and Cell Biology, Chinese Academy of Sciences, Shanghai 200031, China; Shanghai Institute of Nutrition and Health, Chinese Academy of Sciences, Shanghai 200031, China; Department of Nutrition and Food Hygiene, School of Public Health, Institute of Nutrition, Fudan University, Shanghai 200032, China; Key Laboratory of Systems Biology, Center for Excellence in Molecular Cell Science, Shanghai Institute of Biochemistry and Cell Biology, Chinese Academy of Sciences, Shanghai 200031, China; Key Laboratory of Systems Health Science of Zhejiang Province, School of Life Science, Hangzhou Institute for Advanced Study, University of Chinese Academy of Sciences, Hangzhou 310024, China; Department of Nephrology, Longhua Hospital, Shanghai University of Traditional Chinese Medicine, Shanghai 200030, China; Department of Nephrology, Longhua Hospital, Shanghai University of Traditional Chinese Medicine, Shanghai 200030, China; BYHEALTH Institute of Nutrition & Health, Guangzhou 510799, China; BYHEALTH Institute of Nutrition & Health, Guangzhou 510799, China; BYHEALTH Institute of Nutrition & Health, Guangzhou 510799, China; Department of Nephrology, Longhua Hospital, Shanghai University of Traditional Chinese Medicine, Shanghai 200030, China; Key Laboratory of Systems Biology, Center for Excellence in Molecular Cell Science, Shanghai Institute of Biochemistry and Cell Biology, Chinese Academy of Sciences, Shanghai 200031, China; Shanghai Institute of Nutrition and Health, Chinese Academy of Sciences, Shanghai 200031, China; BYHEALTH Institute of Nutrition & Health, Guangzhou 510799, China; Key Laboratory of Systems Health Science of Zhejiang Province, School of Life Science, Hangzhou Institute for Advanced Study, University of Chinese Academy of Sciences, Hangzhou 310024, China; Key Laboratory of Systems Biology, Center for Excellence in Molecular Cell Science, Shanghai Institute of Biochemistry and Cell Biology, Chinese Academy of Sciences, Shanghai 200031, China; School of Life Science and Technology, ShanghaiTech University, Shanghai 201210, China; Shanghai Institute of Nutrition and Health, Chinese Academy of Sciences, Shanghai 200031, China; Key Laboratory of Systems Health Science of Zhejiang Province, School of Life Science, Hangzhou Institute for Advanced Study, University of Chinese Academy of Sciences, Hangzhou 310024, China

**Keywords:** metabolic health, two-dimensional, homeostatic resilience

## Abstract

Defining metabolic health is critical for the earlier reversing of metabolic dysfunction and disease, and fasting-based diagnosis may not adequately assess an individual's metabolic adaptivity under stress. We constructed a novel Health State Map (HSM) comprising a Health Phenotype Score (HPS) with fasting features alone and a Homeostatic Resilience Score (HRS) with five time-point features only (*t* = 30, 60, 90, 180, 240 min) following a standardized mixed macronutrient tolerance test (MMTT). Among 111 Chinese adults, when the same set of fasting and post-MMTT data as for the HSM was used, the mixed-score was highly correlated with the HPS. The HRS was significantly associated with metabolic syndrome prevalence, independently of the HPS (OR [95% CI]: 0.41 [0.18, 0.92]) and the mixed-score (0.34 [0.15, 0.69]). Moreover, the HRS could discriminate metabolic characteristics unseparated by the HPS and the mixed-score. Participants with higher HRSs had better metabolic traits than those with lower HRSs. Large interpersonal variations were also evidenced by evaluating postprandial homeostatic resiliencies for glucose, lipids and amino acids when participants had similar overall HRSs. Additionally, the HRS was positively associated with physical activity level and specific gut microbiome structure. Collectively, our HSM model might offer a novel approach to precisely define an individual's metabolic health and nutritional capacity.

## Teaser

The Health State Map (HSM), which integrates the fasting-based ‘Health Phenotype Score (HPS)’ and postprandial-based ‘Homeostatic Resilience Score (HRS),’ offers a superior approach to the 1D mixed-score framework in depicting metabolic health.

## INTRODUCTION

Metabolic health, which goes far beyond the absence of disease, is a comprehensive state of well-being [1]. Owing to a lack of specific quantification criteria or means, an individual's metabolic health status is routinely defined by using clinical disease-based diagnoses. However, the drawbacks of this approach are that it is almost exclusively based on fasting clinical biomarkers whereas people in the real world remain in a postprandial situation for the majority of the day. Previous studies have shown that postprandial biomarkers such as glucose and triacylglycerol (TAG) levels are independent risk factors for metabolic diseases such as cardiovascular disease (CVD) and type 2 diabetes (T2D), even when these clinical biomarkers are within the normal fasting range [2–5]. Thus, the use of innovative concepts and strategies to redefine metabolic health is urgently needed to discover an earlier trajectory of increased metabolic risk and provide more targeted nutritional interventions.

Recently, ‘homeostatic resilience’, which reflects an individual's ability to adapt to external interruption(s) such as meal intake, has been proposed as an important health criterion [6] and represents a new approach for holistically defining metabolic health [4,7,8]. To test metabolic adaptability, people's metabolic equilibrium must be interpreted and the oral glucose tolerance test (OGTT) is a good example of how to measure dynamic changes in blood glucose following a 75-g glucose challenge. Clinical data have already demonstrated that the OGTT can provide earlier and more precise predictions than the fasting blood glucose test for evaluating glycemic homeostasis in diabetes diagnosis [9]. However, an OGTT that uses only glucose does not reflect the composition of a mixed meal or the diverse metabolites produced in the human postprandial state [5]. To determine metabolic homeostatic resilience for a more diverse set of nutrients [4,10,11], a standardized mixed macronutrient tolerance test (MMTT, containing glucose, fat and protein) was proposed as a simple nutritional challenge tool [4,10,11]. This pioneering work came from researchers in the Netherlands [10,12]. For instance, they developed the ‘health space’ model by integrating blood metabolomic and organ and tissue functional biomarkers measured before and after a ‘PhenFlex test’ [13]—one type of MMTT—and found this model to be more effective in ranking age- and body mass index (BMI)-matched participants with varying phenotypic flexibility or resilience [12]. However, fasting parameters are known to strongly influence post-MMTT response values [3]. On the other hand, the postprandial response itself could more directly and sensitively reflect an individual's adaptive capability across multiple organs and tissues under stress [13]. It is unclear whether a 1D model that combines fasting and postprandial data would result in the overshadowing of postprandial homeostatic resilience by the fasting status results. Therefore, we hypothesized that a 2D model, which separates fasting and postprandial features, would offer a more comprehensive assessment of metabolic health. In this model, the fasting state is treated as one dimension and the postprandial state is treated as another dimension.

**Figure 1.**
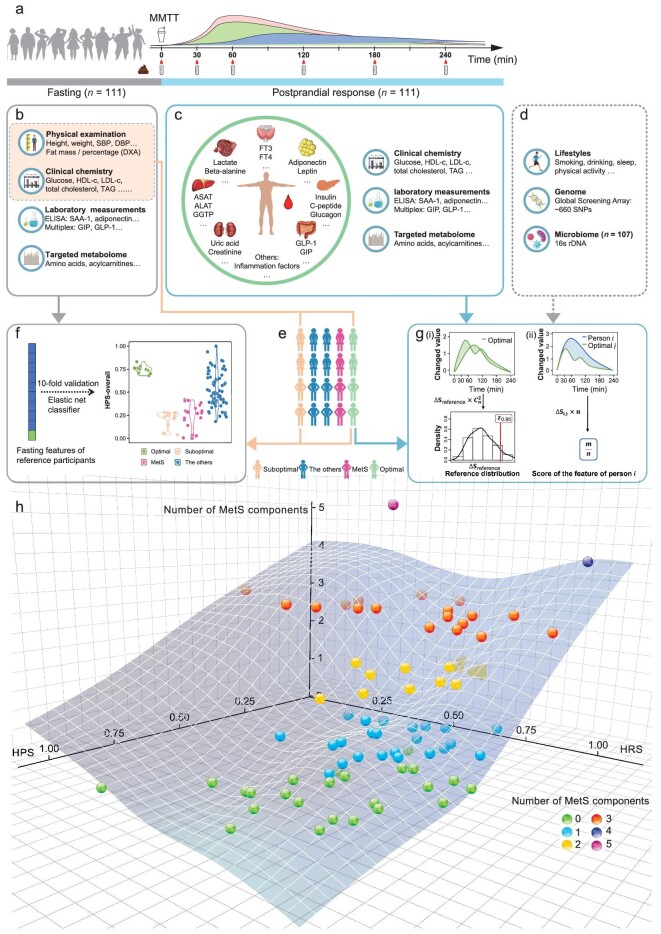
Overview of the study design. (a) MMTT was conducted for the 111 included Chinese adults. (b) The metabolomic and clinical biomarkers and other phenotypes that were exclusively obtained from fasted participants and used to generate HPS or the health space model in (f) are shown. The features in the dashed box were used to select the participants for the two reference groups shown in (e). (c) Metabolomic and clinical biomarkers were measured at five time points post-MMTT treatment. The biomarkers whose levels significantly changed compared with the fasting levels were used to construct the HRS model (g). (d) Factors that may have influenced the HRS are shown. (e) Participants were classified as follows: optimal health reference group (*n* = 11; age <35 years; BMI < 24 kg/m^2^; and clinical blood parameters within normal ranges); suboptimal health reference group (*n* = 15; age >50 years; BMI ≥ 24 kg/m^2^; and without MetS); those with MetS; and others (not in any of the previously described groups). (f) The HPS model was built by using a statistical model named ‘health space’. (g) The HRS model was built via our new ‘ABRC’ model (∆*S_i,j_*: ∆S between person *i* and optimal person *j* (*j* = 1, …, *n*), *m*: the number of ∆S*_i,j_* $> {{z}_{0.95}}$, *n*: the number of optimal persons; for details, see ‘Methods’). (h) Scatter plot of MetS (*z*-axis) with the HPS-overall (*x*-axis) and HRS-overall (*y*-axis) values (*n* = 85). The curve was fitted with components of MetS ∼ f (HPS-overall + HRS-overall). HPS-overall represents the HPS calculated by using all fasting/static features and HRS-overall represents the HRS calculated by using all dynamically changed features post-MMTT. ABRC, area between response curves; DBP, diastolic blood pressure; GIP, gastric inhibitory polypeptide; HPS, Health Phenotype Score; HRS, Homeostatic Resilience Score; MetS, metabolic syndrome; MET-h, metabolic equivalent task hours; MMTT, mixed macronutrient tolerance test (containing 75 g of glucose, 60 g of fat and 20 g of protein in 400 mL of liquid); SBP, systolic blood pressure.

In this study, on the basis of non-linear dynamical theory [14–16], we established a new 2D framework named the Health State Map (HSM) (Fig. 1), comprising the Health Phenotype Score (HPS), which is based on anthropometric parameters and fasting blood metabolomic and clinical organ/tissue functional biomarkers (Fig. 1b), and the Homeostatic Resilience Score (HRS), which is based on dynamic changes in metabolomic and organ/tissue functional biomarkers at five post-MMTT time points (Fig. 1c). We investigated (i) whether the HSM is more informative than a mixed-score with the same set of data, similarly to the ‘health space’ model; (ii) whether the HPS and HRS reflect different aspects of metabolic traits; (iii) whether there was post-MMTT heterogeneity in homeostatic resilience for overall and specific responses to glucose, lipids and amino acids (AAs); and (iv) whether there were potential influences of genetic and non-genetic factors on the HRS (Fig. 1d) among 111 Chinese adults.

## RESULTS

### The baseline characteristics of participants in the HSM group and two reference groups

The baseline characteristics of the 111 sex-matched participants aged 20–70 years in the six subgroups are presented in Table S1. Compared with normal-weight participants of similar ages, overweight/obese participants generally presented lower physical activity levels and poorer cardiometabolic profiles. The baseline characteristics of the two reference groups according to age, BMI and major clinical features (Table S2) are shown in Table S3. As expected, participants in the optimal health group presented significantly lower levels of systolic blood pressure, fasting glucose, HbA1c and TAG than those in the suboptimal health group.

### The 2D model was more informative than the 1D model

**Figure 2.**
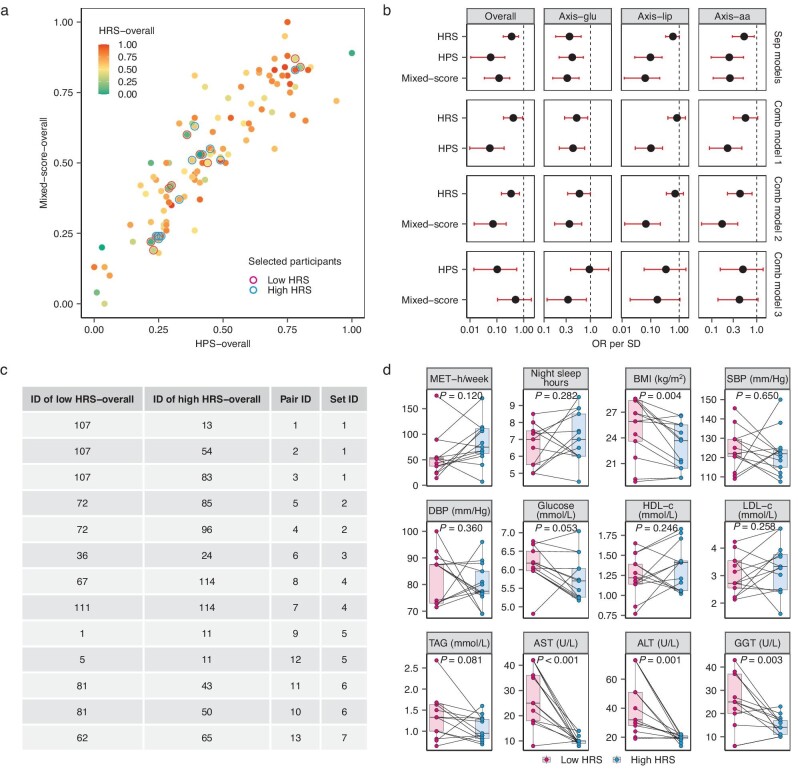
The 2D models containing the HPS and HRS were more informative than the 1D model. (a) Scatterplot of the HPS-overall (*x*-axis), mixed-score-overall (*y*-axis) and HRS-overall (colored dots within the 0–1 scale). (b) Comparison of the associations of MetS status with the HRS (*n* = 100), HPS (*n* = 85) and mixed-score (*n* = 85) separately and in combination. A total of 100 participants were included in the HRS after those assigned to the ‘optimal health’ group were excluded; 85 participants were included in the HPS and mixed-score models after those assigned to the ‘optimal health’ and ‘suboptimal health’ group were excluded. The term ‘combined model’ indicates that all three scores were analysed together via logistic regression. (c) Information of 13 paired selected participants (7 sets). These participants were obtained from (a) and are labeled with circles. Pairs with duplicate participants were grouped into the same set. Each pair had intervals of <0.05 for both the HPS-overall and mixed-score-overall, >0.5 for the HRS-overall and were of the same sex. (d) Characteristics of the selected participants in (c). The *P*-values were calculated by using the score test in conditional logistic regression. aa, amino acid; ALT, alanine transaminase; AST, aspartate aminotransferase; BMI, body mass index; Comb model, combined model; DBP, diastolic blood pressure; GGT, gamma-glutamyl transpeptidase; glu, glucose; HDL-c, high-density lipoprotein cholesterol; HPS, Health Phenotype Score; HRS, Homeostatic Resilience Score; LDL-c, low-density lipoprotein cholesterol; lip, lipid; MET-h, metabolic equivalent task hour; OR, odds ratio; SBP, systolic blood pressure; SD, standard deviation; Sep models, separate models; TAG, triacylglycerol.

The hypothesis of whether a 2D model—the HSM—with a separate HPS for fasting and HRS for post-MMTT changes could provide additional information beyond that of a 1D model such as the mixed-score was examined. As shown in Fig. 2a, when all features in these scores were considered, within the 0–1 scale system from poor to better metabolic health (details in ‘Methods’), the HPS-overall and mixed-score-overall values were strongly correlated with each other (Spearman's *r* = 0.85) but weakly correlated with the HRS-overall value (Spearman's *r* = 0.053 for HPS-overall and *r* = –0.070 for the mixed-score).

The HPS-overall, mixed-score-overall and HRS-overall were inversely associated with metabolic syndrome (MetS) prevalence (OR [95% CI]: 0.06 [0.01, 0.20], 0.15 [0.05, 0.36] and 0.36 [0.17, 0.64], respectively, in Fig. 2b). However, in the combined logistic regression models, the significant association for the mixed-score diminished (Fig. 2b) after adjusting for HPS-overall (0.45 [0.11, 1.94]) whereas the significant association for HRS-overall remained (0.41 [0.18, 0.92], 0.34 [0.15, 0.69]) after adjusting for either the HPS-overall or mixed-score-overall, suggesting that the HRS–MetS association was independent of the scores with fasting or containing fasting features. Moreover, the associations of these three scores with individual metabolic traits were also assessed. A higher HPS-overall and mixed-score-overall were favorably correlated with metabolic traits, as indicated by the significantly higher HDL-c and Matsuda indices but lower fasting glucose, TAG, LDL-c and liver function biomarkers (Fig. S1a and b) compared with those of participants with lower scores. However, almost half of these significant associations were diminished by further adjusting for age and BMI (Fig. S1d and e). On the other hand, a higher HRS-overall was significantly associated with lower body fat percentage and aspartate aminotransferase (AST), leptin and aromatic AA (AAA) levels (Fig. S1c and f) and the associations remained significant even after adjusting for BMI or age.

**Figure 3.**
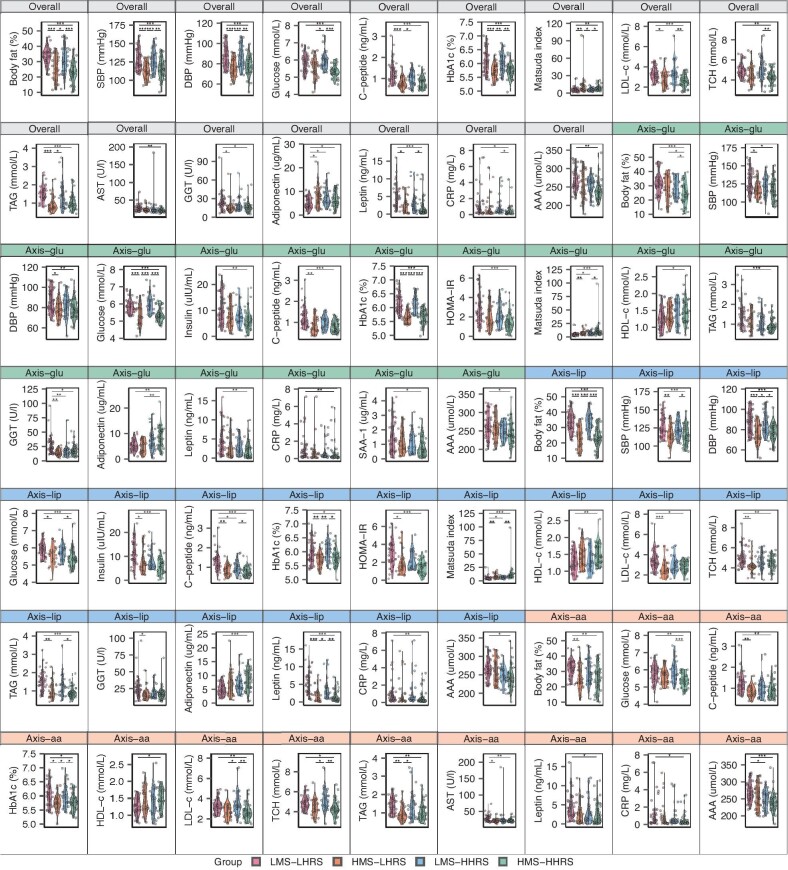
Violin plots comparing single metabolic traits among participants with low HPS + low HRS, high HPS + low HRS, low HPS + high HRS and high HPS + high HRS (*n* = 31, 24, 24 and 32 for overall; 33, 22, 22 and 34 for axis–glu; 33, 21, 22 and 35 for axis–lip; 32, 23, 23 and 33 for axis–aa). Features with FDR-corrected values of <0.05, tested by using analysis of variance or the Kruskal‒Wallis test, are displayed. Post hoc analyses were conducted via Student's *t*-tests or pairwise Wilcoxon signed-rank tests between each pair of groups. FDR was calculated via the Benjamini–Hochberg method to adjust *P*-values for multiple comparisons, with significance levels defined as ***P < 0.001, **P < 0.01 and *P < 0.05. Boxes are shown as medians between the first and third quartiles. aa, amino acid; AAA, aromatic amino acids; AST, aspartate aminotransferase; CRP, C-reactive protein; DBP, diastolic blood pressure; GGT, gamma-glutamyl transpeptidase; glu, glucose; HDL-c, high-density lipoprotein cholesterol; HHPS, high Health Phenotype Score; HHRS, high Homeostatic Resilience Score; LDL-c, low-density lipoprotein cholesterol; LHRS, low Homeostatic Resilience Score; lip, lipid; LHPS, low Health Phenotype Score; SAA-1, serum amyloid protein-1; SBP, systolic blood pressure; TAG, triacylglycerol; TCH, total cholesterol.

To further test whether the HRS per se could discriminate metabolic phenotypes beyond the HPS or the mixed-score, a total of 13 sex-matched pairs of participants with similar HPSs and mixed-scores (<0.05 within the 0–1 score system) but large variance in HRS-overall (>0.5 within the 0–1 score system) were selected (Fig. 2c). Participants with a high HRS-overall had significantly lower BMIs and liver function biomarker levels, including AST, alanine transaminase (ALT) and gamma-glutamyl transpeptidase (GGT) (*P* < 0.05) (Fig. 2d). When all the participants were tested for 24 metabolic traits (details in Table S4) according to whether their three scores were higher or lower than the median, among the participants in the subgroups with low HPSs, those with higher HRSs presented higher adiponectin levels than those with lower HRSs (Fig. 3).

Overall, our results showed that the HSM, as a 2D model, could offer more information for defining metabolic health than a 1D model, such as the mixed-score, even when the same set of fasting and post-MMTT features was used.

### The HRS distinguished interpersonal heterogeneities in response to specific macronutrients during the MMTT

**Figure 4.**
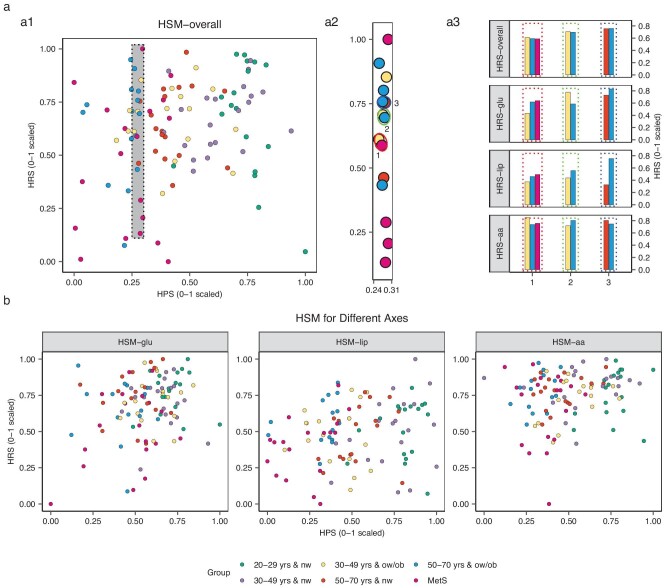
HSM (a) overall and (b) for the three axes (*n* = 111). (a) HPSs were calculated on the basis of participants’ fasting blood metabolomic and clinical features; HRSs were calculated on the basis of solely dynamic changes in metabolomic and clinical biomarkers at five post-MMTT time points after fasting levels were subtracted. (a1) The HPS-overall and HRS-overall for all participants (*n* = 111) are shown on the *x*-axis and *y*-axis, respectively, with both axes scaled from 0 to 1, representing a range from poor to better health status. (a2) An enlarged representation of the dashed box in (a1) including 16 participants within the HPS-overall interval of 0.05 (0.25–0.30) on the *x*-axis who had a large HRS-overall interval of 0.87 (0.13–1.00) on the *y*-axis. (a3) Participants selected from (a2) with a similar HRS-overall (marked by ellipses) are displayed in (a3) showing varying HRSs across different axes. (b) HPSs and HRSs for glucose, lipids and AAs among all participants (*n* = 111) are displayed on the *x*-axis and *y*-axis, respectively, and are scaled from poorer to more favorable health scores (0 to 1). The three axes were determined on the basis of the variables involved in glucose, lipid and AA metabolisms. aa, amino acid; glu, glucose; lip, lipid; HPS, Health Phenotype Score; HRS, Homeostatic Resilience Score; HSM, Health State Map.

As indicated by Fig. 4a1 and a2, at a similar HPS-overall, participants could have a considerably different HRS-overall. For example, within a narrow HPS-overall range (0.25–0.30), the HRS-overall range could vary from 0.13 to 1.00 (17-fold difference), reflecting considerable interpersonal heterogeneity in postprandial homeostatic resilience (Fig. 4a1 and a2). Moreover, participants with a similar HRS-overall presented different postprandial responses to the three macronutrients based on their HRS–glu, HRS–lip and HRS–aa (Fig. 4a3), which were calculated from glucose, lipids and AA metabolism features (see ‘Methods’ and Table S5). This highlights the usefulness of the HRS for distinguishing individuals’ homeostatic capacities for handling macronutrients, which is crucial for optimizing personalized nutrition and targeted interventions.

To determine the responses of features that contributed the most to varied macronutrient resiliencies, HSMs were constructed along three axes and the five most dynamically changed post-MMTT biomarkers on each axis were compared between participants with high and low HRSs (≥median vs. <median values). Participants with high HRSs exhibited (i) smaller increases in glucose levels and larger increases in pyruvate levels for HRS–glu (Fig. S2a); (ii) larger decreases in acylcarnitine levels and smaller increases in TAG levels for HRS–lip (Fig. S2b); and (iii) larger increases in threonine and lysine levels and smaller decreases in aspartate and glutamate levels for HRS–aa (Fig. S2c).

In addition, whether the HRS would offer additional information on three macronutrient axes within the same HPS or mixed-score was also examined via joint analysis. As demonstrated in Fig. 3, compared with those with low HPS and low HRS (both <median scores), participants with ‘low HPS and high HRS’ (≥median) presented a higher Matsuda index but lower GGT concentrations at the HRS–glu axis but lower fasting C-peptide levels and higher Matsuda index values at the HRS–lip axis. On the other hand, compared with those with ‘high HPS and low HRS’, participants with ‘high HPS and high HRS’ had a lower body fat percentage but higher adiponectin levels at the HRS–glu axis. Thus, these findings further support that the post-MMTT feature-based HRS per se could provide more homeostatic insights into glycemic and lipid metabolism beyond the fasting feature-based HPS. Moreover, the results of the mixed-score were similar to those of the HPS (Fig. S3).

### Associations of HSM with lifestyle and genetic factors

**Figure 5.**
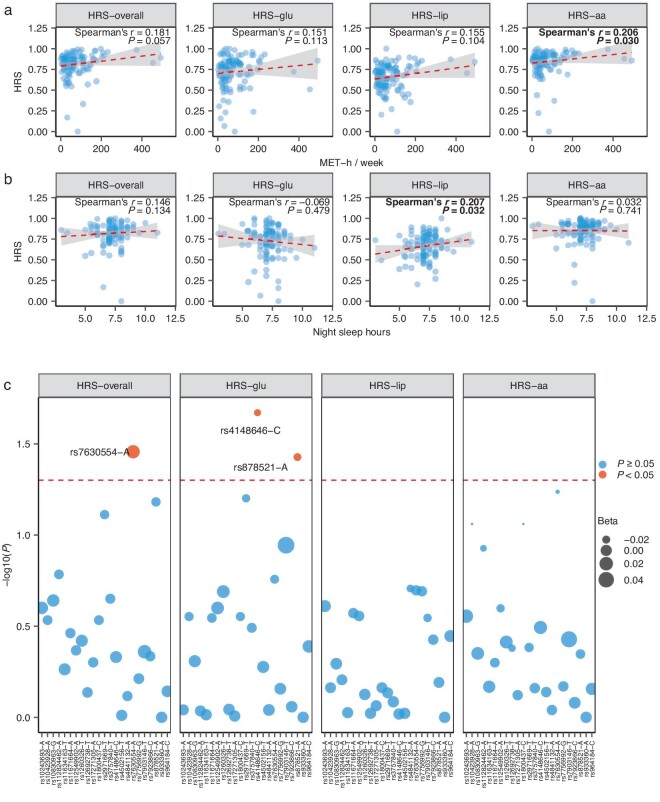
Spearman correlations of the HRS with (a) MET-h, (b) night-time sleep duration and (c) SNPs related to postprandial glucose, insulin or TAG levels (*n* = 111 participants). Separate linear models were used for each SNP. aa, amino acid; beta, standardized regression coefficient for linear model; glu, glucose; HRS, Homeostatic Resilience Score; lip, lipid; MET-h, metabolic equivalent task hours for physical activity levels; Spearman's *r*, Spearman's correlation coefficient.

The impacts of lifestyle on the HSM, HPS and HRS were assessed separately via Spearman's correlations. Physical activity levels were positively correlated with the HPS–lip (*P* < 0.05) (even after adjusting for the HRS–lip (*P* < 0.1, Fig. S4a)), with the HRS–aa (Spearman's *r* = 0.21, *P* = 0.030, Fig. 5a) and, marginally, with the HRS-overall (*r* = 0.18, *P* = 0.057, Fig. 5a). Moreover, night-time sleep duration (range: 3–11 h, interdecile range: 6, 8.5) was also positively correlated with the HRS–lip (Spearman's *r* = 0.21, *P* = 0.032; Fig. 5b). After adjusting for the HPS, these associations remained (Spearman's *r* = 0.18, *P* = 0.075, Fig. S4b). Together, these results suggest that certain lifestyle factors might influence the postprandial resilience of protein and lipid metabolism.

Genetic effects on fasting-based features such as glucose and lipids have been studied extensively but not in the case of postprandial responses. In the present study, 23 single-nucleotide polymorphisms (SNPs) (Table S6) that were reported to modify postprandial responses to glucose, insulin or TAG were selected to evaluate their associations with the HRS via separate linear models. Among them, rs7630554-A in the *IGF2BP2* gene, which is related to reduced 2-h glucose levels after the OGTT, was significantly associated with a higher HRS-overall (*β* = 0.017, *P* = 0.035; Fig. 5c) whereas rs4148646-C in the *ABCC8* gene and rs878521-A close to the *CAMK2B* gene, both of which are related to increased 2-h glucose levels, showed inverse associations with the HRS–glu (*β* = –0.024 and –0.021, respectively; both *P* < 0.05). However, all the significant associations became non-significant after false discovery rate (FDR) correction. Certainly, additional studies with larger sample sizes are needed.

### Associations between the HSM and gut microbiota

**Figure 6.**
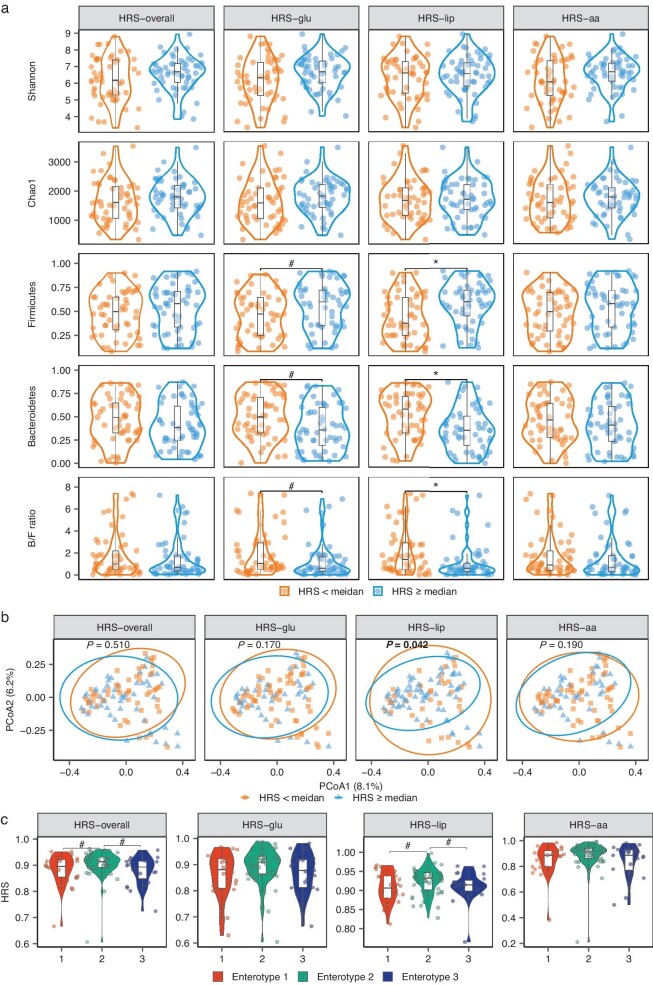
Associations of the gut microbiota structure with the HRS (*n* = 107). Differences in (a) the Shannon and Chao1 indices, the abundance of bacteria in the Firmicutes and Bacteroides phyla, and the Bacteroides/Firmicutes ratio were tested via Wilcoxon tests, and (b) *β* diversity was tested via permutational multivariate analyses of variance between the groups with high (≥median, *n* = 54) and low (<median, *n* = 53) HRSs. (c) Differences in the HRS among the three enterotypes clustered at the genus level were tested via the Jensen‒Shannon distance and the Kruskal‒Wallis test followed by Dunn's post hoc test. Multiple testing was corrected by using Benjamini–Hochberg FDR: # <0.1, * <0.05. Boxes are shown as medians between the first and third quartiles. aa, amino acid; glu, glucose; B/F ratio, ratio of Bacteroidetes/Firmicutes; HRS, Homeostatic Resilience Score; lip, lipid.

The potential effects of gut microbiota on the HPS and HRS were also evaluated in this study. The HPS–overall and HPS–lip but not the HRSs were significantly correlated with alpha diversity, as indicated by the Shannon and Chao1 indices (Fig. S4). Compared with those with a low HRS–lip, participants with a high HRS–lip had a greater abundance of the phylum Firmicutes but a lower abundance of the phylum Bacteroidetes and a lower Bacteroidetes/Firmicutes (FDR < 0.05; Fig. 6a). At the genus level, only *Coprococcus* exhibited a significant positive association with the HRS-overall (FDR < 0.05; Fig. S5). Moreover, significant differences in *β* diversity were also detected between participants with high and low HRS–lip (*P* = 0.042, Fig. 6b). Furthermore, specific associations of certain gut microbiota structures were further evaluated by clustering all participants into three enterotypes via the Jensen‒Shannon distance. Among these three enterotypes, *Prevotella* was dominant in participants with enterotype 1 (mean abundance: 56.8%), *Faecalibacterium* and *Bacteroides* were dominant in participants with enterotype 2 (29.4% and 20.4%, respectively) and *Bacteroides* was dominant in participants with enterotype 3 (59.3%). When participants with enterotypes 1 and 3 were compared, those with enterotype 2 had a higher HRS-overall and HRS–lip (FDR < 0.1, Fig. 6c). Overall, these results indicated that participants with high HRSs, especially a high HRS–lip, might have distinctive gut microbiome structures at the phylum and genus levels.

### Network associations between HRSs with changed single features and conventional metabolic traits

**Figure 7.**
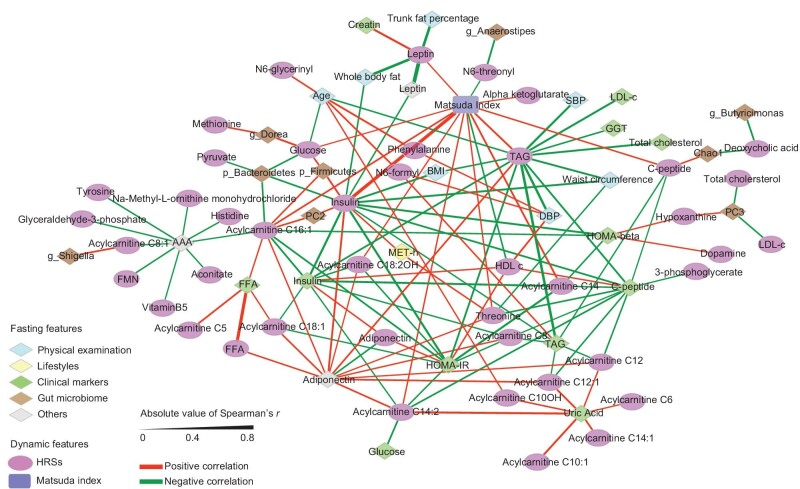
Networks between HRSs of changed single features and metabolic traits (*n* = 111) and microbiome structures (*n* = 107). The FDRs of Spearman's correlations between HRSs and each single feature/metabolic trait were separately estimated via the Benjamini–Hochberg method. Only the features of networks with an FDR of <0.1 are shown. AAA, aromatic amino acid; BMI, body mass index; DBP, diastolic blood pressure; FFA, free fatty acid; FMN, flavin mononucleotide; HDL-c, high-density lipoprotein cholesterol; LDL-c, low-density lipoprotein cholesterol; MET-h, metabolic equivalent task hour; PC, principal component at the microbiota genus level; SBP, systolic blood pressure; TAG, triacylglycerol.

In addition to collective metabolic features, the associations between the HRSs of single features and *β*-cell function, such as the Matsuda index, and other fasting-based metabolic traits were also explored in the current study. Notably, in addition to post-MMTT HRSs for changed insulin, HDL-c or TAG levels, HRSs for changed levels of six medium- to long-chain acylcarnitines (C8, C14, C14:2, C16:1, C18:1 and C18:2OH) were inversely correlated with insulin resistance, as indicated by using homeostasis model assessment of insulin resistance (HOMA-IR) (Fig. 7; Spearman's *r* from –0.43 to –0.28) whereas the HRSs for changed levels of four acylcarnitines (C14, C14:2, C16:1 and C18:2OH) were positively correlated with the Matsuda index (Spearman's *r* from 0.26 to 0.38). Moreover, the HRSs for changed glucose, insulin, C-peptide, TAG or threonine levels were positively associated with the Matsuda index. In addition, the physical activity level was positively associated with the HRS for changed insulin and threonine levels (both Spearman's *r* = 0.31). Thus, our results suggest that the postprandial homeostatic resilience of certain metabolites or clinical biomarkers, particularly changes in medium- to long-chain acylcarnitines post-MMTT, might be essential determinants that are linked with glucose and lipid metabolism.

## DISCUSSION

To our knowledge, this is the first study to show that the HSM—a 2D model—with separated fasting feature-based HPSs and post-MMTT feature-based HRSs is more informative for defining metabolic health than the mixed-score system, even when the same set of features is used among 111 Chinese individuals aged 20–70 years. The HRS could distinguish participants’ metabolic features unseparated by the HPS or the mixed-score and revealed heterogeneous responses for glucose, lipids and AA at the same overall homeostatic resilient scores. Both the HPS and the HRS might be modified by physical activity and specific gut microbiome structures. Thus, our novel HSM might offer a more comprehensive, earlier and more sensitive alternative to define metabolic health and nutritional capacity, which is useful for future clinical applications.

One of the major findings of our study was that the information provided by the mixed-score with all six time points of fasting and post-MMTT features largely overlapped with the HPS that contained fasting features only because both scores were highly correlated and had similar associations with metabolic traits. On the other hand, the HRS with features of five time points post-MMTT was significantly associated with MetS prevalence independently of the HPS and mixed-score, and it could also distinguish certain phenotypes, such as liver function biomarkers AST, ALT and GGT, which were not separated by these two scores (Fig. 2d). Although the underlying biological mechanism(s) are not fully understood, previous studies have suggested that the postprandial state could be influenced by the fasting state [3] and individuals’ capacity to adapt to meals or other environmental disturbances has been recognized as a key feature of metabolic health [17,18], although quantifying such capacity remains a major challenge. There is growing evidence that postprandial hyperglycemia and TAG levels are reported to increase the risk of developing CVD, coronary heart disease and cardiovascular mortality, even when individuals are within normal ranges while fasting [3–5]. Collectively, our findings highlighted that the 2D HSM might provide a more holistic tool to comprehensively and dynamically define metabolic health and reveal earlier signs of impaired organ function or dysmetabolism.

An understanding of interpersonal variations in response to food or meals is critical to formulate precise nutritional recommendations or intervention strategies. To date, it is still debatable whether a high-carbohydrate or high-fat diet is better for weight control and metabolic health [19–21] and one reason is the lack of a means to assess individuals’ capacities to cope with different macronutrients simultaneously. Zeevi *et al*. reported that consumption of the same meal and food resulted in different glucose responses on the basis of continuous glucose monitoring measurements [22]. By comparing three macronutrient axes in addition to the overall HRS (HRS-overall), we documented large interpersonal deviations for the HRS–glu, HRS–lip and/or HRS–aa (Fig. 4a). For example, compared with participants with low scores on three axes, participants with a high HRS–glu presented smaller glucose fluctuations along with higher Matsuda indices but lower GGT levels; participants with a high HRS–lip had smaller TAG fluctuations and greater decreases in acylcarnitine levels; and participants with a high HRS–aa presented greater increases in threonine and lysine levels and smaller reductions in aspartate and glutamate levels. A plausible explanation could be that the participants with high HRSs might have had favorable homeostatic resilience for specific macronutrients with better adaptive functions, such as the liver and *β*-cell mitochondria [23,24]. Taking these findings together, this new framework might offer a potentially useful approach to distinguish individuals’ personal metabolic capacities to metabolize carbohydrates, fat and protein in the content of a given food or meal in the future.

As an adaptive sign of metabolic properties and organ/tissue functions [6], homeostatic resilience can be modified by genetics, lifestyle and the gut microbiome, but available data are scarce. Owing to the limited sample size, we did not detect significant associations between the selected SNPs and the HRS. On the other hand, higher physical activity levels were significantly associated with higher HRS-overall and HRS–aa. The strong association of physical activity with HRS–aa might reflect muscle-related regulation of AA metabolism [25,26]. Moreover, adequate sleep duration at night was associated with a high HRS–lip. Previously, shorter night-time sleep duration was reported to be associated with elevated levels of very low-density lipid cholesterol and TAG [27] whereas either <5–6 or >8–9 h of night-time sleep duration could predict the onset of CVD [28]. In addition, a high ratio of Bacteroides/Firmicutes was shown to be associated with deteriorated lipid homeostatic resilience. In a 6-month full-feeding trial among Chinese individuals, the Bacteroides/Firmicutes ratio was significantly associated with C-reactive protein (CRP) levels [19]. In this study, we also observed that, within the high-HRS-related enterotype-2 classification, four genera—*Faecalibacterium, Coprococcus, Roseburia* and *Bifidobacterium*—had significantly greater abundances (Fig. S6) and these genera were reported to be associated with reduced T2D risk [29,30]. Interestingly, three of these genera (not *Bifidobacterium*) are butyrate producers [31,32] and increased butyrate production could improve the post-OGTT insulin response [33]. Nonetheless, additional studies are needed to clarify the roles of genetic and lifestyle factors and the gut microbiota in modulating homeostatic resilience.

Similarly to the collective features in the HRSs, a single changed feature at post-MMTT could also behave differently from its fasting state. For example, high fasting levels of acylcarnitines, particularly medium- to long-chain acylcarnitines (carbon chains 7–14 and ≥16, respectively), were previously found to be associated with high insulin resistance [34] and incident T2D [35,36] in our study and others. Consistently, we also observed positive associations between the HOMA-IR and fasting levels of C18:1 and C18:2OH (*P* < 0.05). However, greater declines in these two types and other medium- and long-chain acylcarnitines, as the top post-MMTT changed varieties, were associated with a lower HOMA-IR but a higher Matsuda index (Fig. S7). In accordance with our findings of reduced levels of certain acylcarnitines following MMTT, a previous study showed that the consumption of a high-fat (50% energy) meal resulted in significantly decreased postprandial acylcarnitine levels, and a lower nadir of those acylcarnitine levels was significantly linked with lower fatty acid flux and greater insulin sensitivity [37]. Acylcarnitines are known to play a key role in transporting long-chain fatty acids across the inner mitochondrial membrane for *β*-oxidation and can reflect mitochondrial stress [34,38]. Therefore, reduced postprandial acylcarnitine levels might mirror mitochondrial adaptation during the fasting-fed transition and serve as early dynamic biomarkers of mitochondrial dysfunction.

To our knowledge, this is the first study in which a 2D HSM was established to define metabolic health with a fasting-feature-based HPS and postprandial-feature-based HRS. The key strengths of this study include the following: (i) compared with the 1D mixed-score with the same data set, the findings from our study show that the 2D HSM model could more comprehensively and sensitively define metabolic health and early signs of metabolic dysfunctions; (ii) by including three additional macronutrient axes in the HSM, particularly at a given HRS, we uncovered interpersonal variations in response to glucose, lipids and AAs in the content of MMTT; and (iii) we explored the potential impacts of genetic and lifestyle factors and the gut microbiome on homeostatic resilience. As an exploratory study, this study has several limitations. First, the sample size was relatively small but was calculated on the basis of previous studies with similar designs [12]. Second, the HSM was constructed on the basis of the MMTT—an acute tolerance test—in Chinese participants and should be confirmed and improved by more future randomized controlled trials and longitudinal studies in different ethnic groups. Third, given the lack of criteria for postprandial responses, we had to use only the optimal health reference for the HRS, unlike the HPS, which includes both optimal and suboptimal reference groups according to the diagnoses for ‘healthy’ or ‘unhealthy’ states. Finally, to compare our 2D HSM with the 1D ‘health space’ model, we used the same panel of metabolites as reported by van den Broek [12]. Admittedly, multi-omics analysis is needed to better define metabolic health in future studies.

## CONCLUSION

Overall, the findings of this proof-of-concept study highlighted that the HSM, particularly with the HRS, might enhance the current diagnostic capability to detect dysmetabolism and impaired individuals’ adaptability in response to dietary challenges or specific macronutrients. While waiting for more future validation and modification, this 2D HSM might offer a novel and non-disease-based framework to precisely define metabolic health and nutritional function for early and targeted disease intervention.

## METHODS

### Study population and data collection

In this proof-of-concept study, we recruited 111 sex-matched eligible participants (Table S1) aged 20–70 years with normal weight (18.5 kg/m^2^ ≤ BMI <24 kg/m^2^) [39], overweight/obesity (BMI ≥ 24 kg/m^2^) or MetS [39]. All the subjects underwent an oral MMTT after overnight fasting (Fig. S8). Blood samples were collected at fasting and five time points after MMTT (*t* = 0, 30, 60, 120, 180 and 240 min) to measure clinical biomarkers, organ functional biomarkers and targeted metabolites. Other data were collected via questionnaires, physical examinations, DNA phenotyping and 16S rDNA sequencing (details are provided in the Supplementary methods).

### Model construction

#### Defining reference groups

Two reference groups were defined as the ‘optimal health’ reference including participants aged 20–35 years with normal weight and normal ranges of clinical parameters; and the ‘suboptimal health’ reference including participants aged 50–70 years with overweight/obesity but without MetS (Supplementary Methods and Table S2).

#### The HSM

The HSM consists of an HPS and an HRS (Fig. 1) and was constructed as follows:

- The HPS
  The overall HPS (HPS-overall) was constructed by using all 157 fasting-based features (Fig. S9a) mainly according to a previously published study [40]. The model was trained as a classifier to discriminate the two reference groups by using all these features (standardized as *z* scores) through 10-fold cross-validation of elastic net regression (*α* = 0.5). The optimal and suboptimal health reference groups were defined as 1 and 0, respectively. The best prediction model was determined based on the lambda.min parameter whereas the quality of the model was determined by using the deviance parameter (package ‘glmnet’, R version 4.1.3). Finally, the elastic net model produced a regression equation and each participant's HPS-overall was consequently calculated via the corresponding equation.
- The HRS
  The overall HRS (HRS-overall) was built by using all significantly changed post-MMTT features (*n* features = 101) (Table S5) with our newly developed method termed the ‘area between response curves’ (ABRC). The changed features were determined according to whether they were significantly different from their fasting levels at any post-MMTT time point. The ABRC method was proposed to quantify an individual's homeostatic resilience. Presumably, the ‘optimal health’ reference group also had optimal homeostatic resilience and their response curves were considered the reference curves. Therefore, a given person's homeostatic resilience was evaluated by the area (∆*S*) between his or her response curve and the optimal health reference curve. For each feature, a smaller ∆*S* meant that the person was closer to having optimal homeostatic resilience or vice versa. The detailed ABRC method and construction steps are presented in Supplementary Methods and Fig. S10. Briefly, (i) for feature *g* of person *i*, a post-MMTT response curve was formed by plotting the value of *g* at five time points after its corresponding fasting value was subtracted; (ii) the ∆*S* between the curves of each pair of individuals within the optimal health group (*n* = 11) was computed to form a reference ∆*S* vector containing $C_n^2$ elements; (iii) the reference distribution was constructed with the reference ∆*S* vector and the 95th percentile (${{z}_{0.95}}$) of the reference distribution was calculated; and (iv) the *n* ∆*S* values between the response curves of person *i* and the optimal health reference persons were calculated (*n* = 11) and ∆*S* > ${{z}_{0.95}}$ was considered significant. The HRS of this feature was calculated as (*n* – *m*)/*n* where *m* was the number of significant ∆*S* values and (v) according to Steps (i)–(iv), the HRS of each feature of person *i* was calculated. The HRS-overall was computed as the mean of the HRSs of all 101 features.

#### Mixed-score

The overall mixed-scores (mixed-score-overall) were constructed by integrating all pre- and post-MMTT features (Fig. S9a) via the ‘health space’ modeling method [41].

#### Setting up metabolic axes

In addition to the HPS-overall, HRS-overall or mixed-score-overall, each of these scores was further evaluated by constructing three axes, namely glucose (axis–glu), lipids (axis–lip) and AAs (axis–aa), to evaluate the individuals’ metabolic capacities for glucose, lipids and AAs by modeling biologically relevant metabolites and clinical biomarkers according to the KEGG and HMDB databases or previously published work [12] (Fig. S9b for HPSs and mixed-scores and Table S5 for HRSs).

## Supplementary Material

nwae425_Supplemental_File
