# Supplementary material for: Two-dimensional Health State Map to define metabolic health using separated static and dynamic homeostasis features: a proof-of-concept study
Source: Natl Sci Rev. 2024 Nov 26;12(1):nwae425. doi: 10.1093/nsr/nwae425 (PMC11734281; doi:10.1093/nsr/nwae425)
Supplement: nwae425_Supplemental_File [file nwae425_supplemental_file.docx]

**Supplementary Materials**

**Supplementary Methods**

1. **Study design**

In this proof-of-concept study, we constructed a two-dimensional model with separated fasting and post mixed macronutrient tolerance test (post-MMTT) parameters to define metabolic health by conducting an open-label acute intervention trial at Longhua Hospital in Shanghai, China. The study protocol (ClinicalTrials.gov: NCT04173728) was approved by the institutional review boards of the Shanghai Institutes for Biological Sciences, Chinese Academy of Sciences and Longhua Hospital, Shanghai University of Traditional Chinese Medicine. A written informed consent was obtained from each participant.

1. **Participants**

A total of 149 volunteers were recruited *via* advertisement, and 114 sex-matched eligible participants were assigned to one of the following six groups: 1) aged >20-29 years with a normal weight (18.5 kg/m^2^ ≤ BMI < 24 kg/m^2^)[1]; 2) aged 30-49 years with a normal weight; 3) aged 30-49 years with overweight/obese (BMI ≥ 24 kg/m^2^)[1]; 4) aged 50-70 years with a normal weight; 5) aged 50-70 years with overweight/obese; and 6) aged 30-70 years with metabolic syndrome (MetS). The exclusion criteria included: 1) clinical diagnosis of diabetes or use of antidiabetic medications; 2) clinical diagnosis of cardiovascular, kidney, liver, pituitary, alimentary tract, or thyroid diseases, cancer(s), or mental illnesses; 3) pregnancy or lactation; 4) having undergone gastrointestinal surgery within 1 year, except for appendicitis or hernia,; 5) current use of antidepressant(s); 6) alcohol consumption > 40 g/d or other substance use; 7) severe diarrhoea (watery stools ≥ 3 times/day × 3 days or longer) in the previous 3 months; or 8) participation in any other clinical study within previous 3 months. All 114 eligible participants underwent a standard MMTT, and three of them were withdrawn due to unsuccessful blood collection. A total of 111 participants were included in the final analyses (**Fig. S8**).

1. **Acute intervention and data collection**

After fasting for ≥ 10 hours, all participants underwent oral MMTT. Following the consumption of a 400 ml beverage (~3950 kJ) containing 75 g glucose, 60 g fat, and 20 g protein [2], blood samples were collected *via* a catheter in fasting state (*t* = 0 min) and at 5 successive time points post-MMTT (*t* = 30, 60, 120, 180, and 240 min). A total of 107 participants also provided faecal samples collected in commercial faecal preservation tubes (Shanghai Ruiyi Biotechnology Co., Ltd.).

A face-to-face interview was conducted by trained dieticians with a standardized questionnaire to obtain information about demographic characteristics, lifestyle factors, night-time sleep duration, medical history, and medication use[3]. Physical activity was assessed by the short-form of the International Physical Activity Questionnaire with minor modifications[4], and weekly metabolic equivalent task hours (MET-h) were calculated as the MET-h coefficient of activity × duration (hours) × frequency (days)[4]. The MET-h coefficients of 2.5, 4.0, 8.0, and 3.3 corresponded low, moderate, and vigorous activity levels and walking, respectively. Current smoking and drinking status were defined as yes or no. Body weight, height, waist circumference, and blood pressure were measured by a standardized protocol[3]. BMI was calculated as kg/m^2^, normal weight and overweight/obese status were defined based on the Chinese criteria[1]. In addition, whole-body and trunk fat/fat percentages were measured *via* dual-energy X-ray scan (DXA, QDR-4500; Hologic, Waltham, MA, USA).

1. **Blood sample processing and laboratory measurements**

Blood samples were collected in tubes containing either a clot activator or ethylenediaminetetraacetic acid (K2EDTA) for serum and plasma isolation, respectively. The dipeptidyl-peptidase IV (DPPIV) inhibitor aprotinin and serine protease inhibitor were added to the tubes immediately following blood collection to measure glucagon-like peptide-1 (GLP-1), glucagon, and active ghrelin levels.

Blood samples were centrifuged at 4°C and stored at -80°C until analysis. Serum glucose, insulin, triacylglycerol (TAG), total cholesterol (TCH), high-density lipoprotein cholesterol (HLD-c), low-density lipoprotein cholesterol (LDL-c), free triiodothyronine (FT3), free thyroxine (FT4), thyroid-stimulating hormone (THS), C-reactive protein (CRP), gamma-glutamyl transpeptidase (GGT), alanine aminotransferase (ALT), aspartate aminotransferase (AST), free fatty acid (FFA), and creatinine levels were measured by an automatic analyser (Hitachi 7080) using commercial kits from Wako Pure Chemical Industries. Erythrocyte HbA1c levels were measured by immunoturbidimetry on an automatic analyser (Roche Cobas C311). The plasma adiponectin, E-selectin, P-selectin, human serum amyloid A protein-1 (SAA-1), intercellular cell adhesion molecule-1 (ICAM-1), and vascular cell adhesion molecule-1 (VCAM-1) levels were determined *via* enzyme-linked immunosorbent assay (ELISA). The plasma C-peptide, ghrelin, gastric inhibitory peptide (GIP), GLP-1, glucagon leptin, interferon-γ (IFN-γ), interleukin-1β (IL-1β), IL-6, IL-8, IL-18, IL-10, tumour necrosis factor-α (TNF-α), and vascular endothelial growth factor A (VEGFA) levels were measured using a MILLIPLEX assay kit (Millipore Sigma).

The homeostatic model assessment of insulin resistance (HOMA-IR) score[5] was calculated as fasting glucose (mmol/L) × fasting insulin (mmol/L)/22.5. The homeostatic model assessment of β-cell function (HOMA-B) score[5] was calculated as 20 × fasting insulin (μU/mL)/(fasting glucose [mmol/L] − 3.5). Insulin sensitivity indicated by the Matsuda index was calculated as 10,000/√[(fasting insulin (mU/L) × fasting glucose (mg/dL)) × (mean MMTT insulin (mU/L) × (mean MMTT glucose (mg/dL))][6]. The estimated glomerular filtration rate (eGFR, mL/min per 1.73 m2) was calculated as 175 × creatinine (mg/dL) -1.234 × age-0.179 × 0.79 (if female)[7].

1. **Targeted metabolomic profiling**

Targeted metabolomics was performed on a Shimadzu Nexera X2 LC-30AD ultra-performance liquid chromatograph. A Waters ACQUITY UPLC BEH Amide Column (130 Å, 1.7 µm, 2.1 mm × 100 mm) was used with an elution flow rate of 300 µL/min and a linear gradient of 20 min. Mobile phase A consisted of 5:95 (v/v) acetonitrile/water with 20 mM ammonium acetate (pH 9.0 adjusted with ammonium hydroxide), and mobile phase B was acetonitrile. The chromatographic gradient program was as follows: mobile phase B was started at 95% for 1 min, decreased linearly to 65% for 13 min, followed by a linear gradient reduction to 35% for 1 min; this level was maintained for 2 min; and, finally, the column was equilibrated at 85% for 3 min. Mass spectrometry data were acquired using a hybrid triple quadrupole/linear ion trap mass spectrometer (SCIEX 5500 QTRAP). The operational parameters were set as follows: curtain gas, 35.0; collision gas, medium; temperature, 500°C; ion source gas 1, 40; and ion source gas 2, 50. The ion spray voltages were 5500 and -4500 in positive and negative modes, respectively. Quantitation was performed based on the ratios of the peak areas of the analytes and their corresponding stable isotope-labelled internal standards using MultiQuant™ 3.0 Software (Sciex, Foster City, CA). The plasma samples were analysed in a random order, and quality control samples were measured every 12 samples to ensure repeatability.

The sum of fasting L-histidine, L-phenylalanine, and L-tyrosine levels was used to calculate the amount of plasma aromatic amino acids (AAAs); the sum of fasting L-isoleucine, L-leucine, and L-valine levels was used to determine branched-chain amino acid (BCAA) levels; and the sums of fasting levels of L-alanine, L-arginine, L-asparagine, L-aspartic acid, L-cysteine, glutamic acid, L-glutamine, L-glycine, L-histidine, L-methionine, L-proline, L-serine, and L-valine were used to determine specific glucogenic amino acid levels.

1. **SNP genotyping**

DNA was extracted from peripheral blood leukocytes using a QIAamp DNA Kit (Qiagen, Hilden, Germany) and then the DNA was used for genotyping with an Infinium Global Screening Array-24 v3.0 Kit. The data were excluded if a SNP had a call rate < 95%, had a minor allele frequency (MAF) < 1%, or was not in Hardy‒Weinberg equilibrium (*P* < 10^-6^). Ungenotyped or missing SNPs were imputed based on the 1000 Genomes haplotype reference panel (phase 3) with IMPUTE version 2.1.2 software. Imputed SNPs were removed when the estimated call rate was < 95%, the MAF was < 1%, or the Hardy‒Weinberg equilibrium (P) value was < 10-6. In addition, the SNPs related to postprandial responses to glucose[8], TAG[9], or insulin[10] in previous genome-wide association studies were extracted from the imputed genome-wide genotypes by using PLINK software.

1. **Microbiome 16S rDNA gene sequencing**

Faecal DNA was extracted using a QIAamp DNA Stool Mini Kit (Qiagen, Hilden, Germany). The V3–V4 region of the bacterial 16S rRNA gene was amplified by polymerase chain reaction (PCR) with the forwards primer 338F (5'-ACTCCTACGGGAGGCAGCA-3') and the reverse primer 806R (5'-GGACTACHVGGGTWTCTAAT-3'). The PCR amplicons were subsequently purified using Vazyme VAHTSTM DNA Clean Beads (Vazyme, Nanjing, China) and quantified using the Quant-iT PicoGreen dsDNA Assay Kit (Invitrogen, Carlsbad, CA, USA). After quantification, individual amplicons were pooled in equal amounts, and paired-end 2 × 250 bp amplicons were sequenced using the Illumina MiSeq Reagent Kit v3 and the MiSeq platform. Microbiome bioinformatics of the raw sequence data was performed using QIIME2 2019.4 as described in official tutorials, with slight modifications[11]. Briefly, raw sequence data were demultiplexed using the demux plugin, and primers were trimmed using the Cutadapt plugin[12]. Subsequently, the sequences were merged, quality filtered, and dereplicated using the Vsearch plugin. All the unique sequences were subsequently clustered at 98%, followed by chimeric sequence removal. Finally, the nonchimeric sequences were reclustered at 97% to generate operational taxonomic unit (OTU) representative sequences and an OTU table.

1. **Definition of metabolic syndrome**

MetS was defined based on the updated National Cholesterol Education Program Adult Treatment Panel III criteria for Asian-Americans[13]. Participants were considered to have MetS if they had three or more of the following features: 1) a waist circumference ≥ 90 cm in men or ≥ 80 cm in women; 2) a TAG level ≥ 1.7 mmol/L; 3) an HDL-c level < 1.03 mmol/L in men or < 1.30 mmol/L in women; 4) an SBP ≥ 130 mm Hg or DBP ≥ 85 mm Hg or current use of antihypertensive medications; and 5) a fasting glucose level ≥ 5.6 mmol/L, previously diagnosed T2D, or treatment with oral antidiabetic agents.

1. **Health State Map (HSM)**

To swift the current disease-centric definition to more physiological-based definition of metabolic health, the nonlinear dynamical theory[14-16] was used to construct our two-dimensional HSM comprising the health phenotype score (HPS) for fasting metabolomic and clinical features and the post-MMTT homeostatic resilience score (HRS) for dynamically changing metabolomic and clinical features. Accordingly, the energy function or potential of a given biological system can be broken down into one state potential and another resilience potential corresponding to the HPS and HRS, thus providing a theoretical background for the HSM. The steps used for the construction of the HSM framework are as follows:

1. **The HPS**

Details was presented in Methods

1. **The HRS**

**Characterizing homeostatic resilience by ∆S**

For each feature (*g)* in a given participant (*i)*, a response or resilience curve was generated by repeatedly measuring fluctuations in metabolomic and clinical biomarkers at 6 time points (*t* = 0, 30, 60, 120, 180 and 240 min) over the standard oral MMTT (**Fig. S10a**). The resilience curves of the individuals in the predefined optimal health reference group were considered normal reference curves since all participants in this group had clinical phenotypes within normal ranges and were more likely to have optimal homeostatic resilience (**Table S5**). Therefore, each participant’s homeostatic resilience was quantified by the area (∆S) between his or her response curve and the reference curve (**Fig. S10b**). A larger ∆S between the response curves of a given participant and the reference group indicated that the participant had deteriorated homeostatic resilience and *vice versa*. Accordingly, the HRS of the participants was calculated using ∆S for all features.

**Calculating the ∆S between participants for feature g**

To calculate the ∆S between two participants (participants *x* and *y*) for a specific feature *(g)*, the vectors $x_{t}, y_{t} (t=1,2,\ldots,6)$ were used to represent the measured values of feature *g* for participant *x* and participant *y*, and the sampling time point was denoted as the vector $T (T=0, 0.5, 1,2,3,4)$. To reduce the effect of the baseline phenotype on the model, we first normalize the vector $x_{t}, y_{t}$ by equation (1):

$$\begin{aligned} x_{t}^{*}=x_{t}-x_{1}, y_{t}^{*}=y_{t}-y_{1} \left( t=1,2,\ldots,6 \right)\#\left( 1 \right) \end{aligned}$$

where $x_{t}^{*},y_{t}^{*}$ are the normalized measured values of feature *g* for participant *x* and participant *y* for subsequent analyses; second, we calculated the ∆S$(t,t+1)(t=1,2,\ldots,5)$ between the response curves of participant *x* and participant *y* at two adjacent time points by the following equation:

∆$T=T_{t+1}-T_{t},$

∆S$(t,t+1)=\left\{ \begin{aligned} 0.5\times\left| x_{t}-y_{t}+x_{t+1}-y_{t+1} \right|\times\Delta T, &(x_{t}-y_{t})\times(x_{t+1}-y_{t+1})\geq0 \\ 0.5\times\left| \left( x_{t}-y_{t} \right)\times p \right|+0.5\times\left| (x_{t+1}-y_{t+1})\times(\Delta T-p) \right|, &(x_{t}-y_{t})\times(x_{t+1}-y_{t+1})<0 \end{aligned} \right.$

where $(x_{t}-y_{t})\times(x_{t+1}-y_{t+1})\geq0$ indicates that the two response curves did not intersect in the interval $(T_{t},T_{t+1})$, while $(x_{t}-y_{t})\times(x_{t+1}-y_{t+1})<0$ indicates that the two response curves did intersect in the interval $(T_{t},T_{t+1})$ at the intersection $p=\left| (x_{t}-y_{t})\times\Delta T/(x_{t}-y_{t}-x_{t+1}+y_{t+1}) \right|$. Thus, we can obtain the ∆S$(t,t+1)(t=1,2,\ldots,5)$ between any two adjacent time points for all the measured features. Finally, we calculated the ∆S between participant *x* and participant *y* for a given feature *g* by equation (2):

$$\begin{aligned} \Delta S=\sum_{t=1}^{5} \Delta S\left( t,t+1 \right)\#\left( 2 \right) \end{aligned}$$

**Constructing the reference distributions and calculating the 95th percentile**

For feature *g*, first, we calculated the ∆S between each pair of *n* participants in the optimal health reference group to form a reference ∆S vector containing $C_{n}^{2}$ elements (**Fig. S10b**). The reference ∆S vector represents the difference between the participants with optimal homeostatic resilience. Second, we constructed reference distributions with the reference ∆S vector and calculated the 95th percentile ($z_{0.95}$) of the reference distributions (**Fig. S10c**). The reference distribution represents the distribution of differences in curves between participants with optimal homeostatic resilience. The difference in curves between two participants was deemed nonsignificant when ∆S was ≤ $z_{0.95}$, while the difference was considered significant when ∆S was > $z_{0.95}$. The specific process by which $z_{0.95}$ was calculated is as below.

The reference ∆S vector was a set of samples from the reference distribution. To reduce the effect of sample error, we first tested the normality of the reference distribution in the reference ∆S vector (function shapiro.test in R language). *P* was the *P* value of the normality test. Due to the relatively small sample size, normality tests were performed at the 0.01 significance level, with *P* > 0.01 indicating that the tested distribution significantly followed a normal distribution. Then, a reference normal distribution was constructed from the reference ∆S vector, and the 95th percentile ($z_{0.95}$) of the normal distribution was calculated. When *P* was ≤ 0.01, $z_{0.95}$ was simply equal to the 95th percentile of the reference ∆S vector.

**Computing the resilience score of participant *k***

Given a vector $y$ of the measured values at six time points for feature *g* of given participant *k*, the ∆S between the participant and each reference participant in the optimal health reference group was calculated (**Fig. S10d**). Thus, we obtained a vector *x* containing *n* (the number of participants in the optimal health reference group) ∆S elements. The proportion of the elements > $z_{0.95}$ in vector *x* was calculated by $m/n$, where $m$ was the number of the elements > $z_{0.95}$ in vector *x*, and the proportion of the elements ≤ $z_{0.95}$ in vector *x* was calculated by $(n-m)/n$ (**Fig. S10e**)*.* The score $(n-m)/n$ characterizes the resilience of feature *g* of the participant (**Fig. S10f**).

The resilience score of each feature was calculated in the same way. Thus, the mean of the scores for all features, or the features within a specific group, was calculated to determine participant’s resilience scores. A low resilience score reflects greater deviation from the optimal health reference, suggesting less homeostatic resilience in the participant, and *vice versa*.

**Statistical analyses**

Two-tailed Student’s *t* tests or Wilcoxon rank tests were used for continuous variables and χ^2^ tests were used for categorical variables. Mixed-effect models were used to assess the post-MMTT changes. Spearman’s correlation test was used to assess the correlations. Separate linear models were used to determine the associations between the HRS and SNPs. Logistic regression models were used to calculate the odds ratios (ORs) of MetS with the HPSs, HRSs, and mixed-scores. Score tests in conditional logistic regression were used to compare differences between 7 sets (13 pairs) of participants with high and low HRS-overall. ANOVA or the Kruskal-Wallis test was used for multi-group comparisons. Post-hoc analyses were conducted using Student’s *t* tests or pairwise Wilcoxon signed-rank tests for each pair of groups and the Benjamin–Hochberg false discovery rate (FDR) was applied for multiple testing correction. All statistical analyses were conducted using R (version 4.1.3).

Community richness and diversity of gut microbiota were estimated by Chao1 and Shannon indices. PCoA based on Bray‒Curtis distance and PERMANOVA was employed to compare global microbiota composition at the genus level. Enterotypes were determined using the abundances of genera by Jensen‒Shannon distance (using the vegan and ape packages in R version 4.1.3). Differences in HRSs and the abundances of genera among the three enterotypes were tested by the Kruskal‒Wallis test followed by Dunn's post hoc test.

**Table S1 Baseline characteristics of participants in different age, BMI, and MetS groups**

|  | **Total** | **20–29 yrs** | **30–49 yrs** | | **50–70 yrs** | | **MetS** |
| --- | --- | --- | --- | --- | --- | --- | --- |
|  |  | **Normal weight** | **Normal weight** | **Overweight/obese** | **Normal weight** | **Overweight/obese** |  |
| **Number (n)** | 111 | 20 | 21 | 17 | 18 | 15 | 20 |
| **Age, years** | 44 ± 14 | 27 ± 2 | 37 ± 5 | 38 ± 6 | 58 ± 6 | 60 ± 6 | 52 ± 11 |
| **Male, n (%)** | 55 (50) | 10 (50) | 10 (48) | 10 (59) | 8 (44) | 7 (47) | 10 (50) |
| **Current smoker, n (%)** | 18 (16.2) | 2 (10.0) | 3 (14.0) | 4 (24.0) | 2 (11.0) | 2 (13.0) | 5 (25.0) |
| **Current drinker, n (%)** | 68 (62.0) | 13 (68.0) | 13 (62.0) | 11 (65.0) | 11 (61.0) | 8 (53.0) | 12 (60.0) |
| **MET-h/week, h** | 55.5 [29.0, 105] | 65.6 [40.4, 95] | 56.3 [48.6, 109] | 23.3 [15.3, 41.5] | 81.0 [32.3, 124] | 57.0 [37.6, 83.2] | 46.9 [28.6, 118] |
| **Night sleep hours** | 7.17 [7.00, 8.00] | 7.17 [6.75, 7.75] | 7.17 [6.00, 7.50] | 7.50 [7.00, 8.00] | 7.17 [6.62, 8.00] | 7.00 [7.00, 7.25] | 7.25 [7.00, 8.00] |
| **BMI, kg/m^2^** | 23.8 ± 3.28 | 20.8 ± 1.40 | 21.9 ± 1.28 | 26.8 ± 2.28 | 21.9 ± 1.48 | 26.4 ± 2.94 | 26.2 ± 3.09 |
| **Waist circumference, cm** | 81.3 ± 11.0 | 71.1 ± 6.15 | 74.0 ± 6.05 | 89.3 ± 8.08 | 76.1 ± 5.17 | 90.4 ± 9.32 | 90.3 ± 8.85 |
| **Body fat, %** | 28.3 ± 8.20 | 23.1 ± 7.12 | 23.9 ± 8.56 | 30.9 ± 7.12 | 27.4 ± 7.14 | 34.4 ± 6.40 | 31.9 ± 6.55 |
| **Trunk fat, %** | 30.0 ± 8.95 | 22.9 ± 6.54 | 23.4 ± 7.70 | 33.4 ± 6.08 | 29.3 ± 8.31 | 38.0 ± 6.33 | 36.0 ± 6.01 |
| **Lean body mass, kg** | 46.6 ± 8.95 | 44.3 ± 6.80 | 46.5 ± 9.94 | 51.6 ± 7.71 | 41.6 ± 5.59 | 46.6 ± 9.26 | 49.2 ± 10.7 |
| **SBP, mm Hg** | 121 ± 14.4 | 109 ± 11.1 | 115 ± 15.1 | 121 ± 11.4 | 126 ± 10.0 | 119 ± 7.75 | 135 ± 13.4 |
| **DBP, mm Hg** | 81.9 ± 11.1 | 72.2 ± 5.80 | 78.0 ± 11.1 | 84.3 ± 11.1 | 84.0 ± 6.13 | 80.2 ± 6.93 | 92.8 ± 11.3 |
| **Fasting glucose, mmol/L** | 5.65 ± 0.54 | 5.38 ± 0.42 | 5.41 ± 0.56 | 5.5 ± 0.36 | 5.7 ± 0.62 | 5.91 ± 0.52 | 6.04 ± 0.42 |
| **HbA1c, %** | 5.89 ± 0.37 | 5.69 ± 0.17 | 5.73 ± 0.25 | 5.71 ± 0.36 | 6.03 ± 0.32 | 6.17 ± 0.39 | 6.06 ± 0.43 |
| **Fasting insulin, IU/L** | 8.37 ± 5.01 | 7.03 ± 3.80 | 6.85 ± 4.40 | 11.3 ± 5.24 | 6.11 ± 3.85 | 8.35 ± 4.01 | 10.9 ± 6.22 |
| **Total cholesterol, mmol/L** | 4.68 ± 0.91 | 4.26 ± 0.80 | 4.55 ± 0.83 | 4.74 ± 1.34 | 4.95 ± 0.79 | 4.6. ± 0.84 | 4.98 ± 0.70 |
| **HDL-c, mmol/L** | 1.39 ± 0.34 | 1.46 ± 0.28 | 1.56 ± 0.33 | 1.22 ± 0.30 | 1.51 ± 0.33 | 1.39 ± 0.33 | 1.15 ± 0.26 |
| **LDL-c, mmol/L** | 2.96 [2.43, 3.39] | 2.55 [2.25, 2.9] | 2.77 [2.41, 3.19] | 3.14 [2.38, 3.46] | 3.30 [2.95, 3.64] | 3.03 [2.46, 3.44] | 3.14 [2.82, 3.65] |
| **TAG, mmol/L** | 1.02 [0.79, 1.51] | 0.81 [0.67, 0.89] | 0.95 [0.73, 1.02] | 1.02 [0.79, 1.63] | 1.27 [0.97, 1.56] | 1.00 [0.92, 1.30] | 1.81 [1.50, 2.22] |
| **FT3, pmol/L** | 4.88 ± 0.63 | 4.98 ± 0.84 | 4.76 ± 0.72 | 4.81 ± 0.57 | 4.78 ± 0.45 | 4.88 ± 0.47 | 5.08 ± 0.59 |
| **FT4, pmol/L** | 16.1 ± 2.24 | 16.4 ± 2.39 | 16.1 ± 2.58 | 15.7 ± 2.07 | 16.0 ± 1.58 | 15.6 ± 1.68 | 16.5 ± 2.78 |
| **TSH, IU/L** | 2.21 [1.6, 3.29] | 2.26 [1.63, 2.92] | 2.45 [1.4, 3.02] | 2.12 [1.68, 2.51] | 2.48 [1.83, 4.2] | 2.00 [1.6, 3.58] | 2.21 [1.56, 3.04] |
| **AST, U/L** | 22.0 [19.0, 26.5] | 19.5 [17.8, 22.0] | 21.0 [19.0, 26.0] | 21.0 [19.0, 24.0] | 23.0 [20.0, 27.8] | 21.0 [18.0, 28.5] | 26.0 [24.2, 33.2] |
| **ALT, U/L** | 15.0 [10.0, 21.5] | 10.5 [7.75, 13.2] | 14.0 [11.0, 16.0] | 20.0 [12.0, 28.0] | 11.0 [8.25, 19.0] | 14.0 [11.0, 21.0] | 21.0 [18.0, 26.8] |
| **GGT, U/L** | 17.0 [13.0, 22.5] | 14.0 [11.0, 15.5] | 15.0 [13.0, 19.0] | 18.0 [14.0, 27.0] | 17.0 [13.2, 20.0] | 13.0 [11.0, 21.5] | 26.0 [19.8, 34.8] |
| **CRP, mg/L** | 0.40 [0.20, 1.11] | 0.20 [0.10, 0.83] | 0.30 [0.10, 0.50] | 0.60 [0.20, 1.10] | 0.30 [0.20, 1.10] | 0.50 [0.20, 1.05] | 0.65 [0.30, 1.70] |

The data are reported as the mean ± standard deviation or median (1^st^ and 3^rd^ quartiles) for continuous variables and percentages for categorical variables.

Weight classification was performed based on the Chinese criteria for individuals with a normal weight (18.5 kg/m^2^ ≤ BMI < 24 kg/m^2^) and overweight/obese individuals (BMI ≥ 24 kg/m^2^).

ALT: alanine aminotransferase, AST: aspartate aminotransferase, BMI: body mass index, CRP: C-reactive protein, DBP: diastolic blood pressure, FT3: free triiodothyronine, FT4: free thyroxine, GGT: gamma-glutamyl transpeptidase, HDL-c: high-density lipoprotein cholesterol, LDL-c: low-density lipoprotein cholesterol, MET-h: metabolic equivalent task hours, MetS: metabolic syndrome, SBP: systolic blood pressure, TAG: triacylglycerol, TSH: thyroid-stimulating hormone.

**Table S2 Criteria for reference group stratification**

| **Reference group** | **Criteria** |
| --- | --- |
| **Optimal health**  **(n = 11)** | 20 years ≤ age < 35 years |
|  | 18.5 kg/m^2^ ≤ BMI < 24 kg/m^2^ |
|  | Waist circumference < 90 cm for men, < 80 cm for women[13] |
|  | SBP < 130 mm Hg[13] |
|  | DBP < 85 mm Hg[13]  Fasting glucose ≤ 5.6 mmol/L[1] |
|  | Total cholesterol < 5.2 mmol/L[18] |
|  | HDL-c ≥1.03 mmol/L for men; ≥ 1.3 mmol/L for women[13]  LDL-c < 3.4 mmol/L[18] |
|  | TAG < 1.7 mmol/L[13] |
|  | FT3 2.1-5.4 pmol/L[19] |
|  | FT4 9-25 pmol/L[19] |
|  | TSH 0.3-4.50 IU/L[19]  AST 15-40 U/L for men; 13-35 U/L for women  ALT 9-50 U/L for men; 7-40 U/L for women  GGT 8-61 U/L for men; 5-36 U/L for women  eGFR > 90 mL/min per 1.73 m^2^ [20] |
| **Suboptimal health**  **(n = 15)** | Age of 50-70 years |
|  | BMI ≥ 24 kg/m^2^  Without MetS |

ALT: alanine aminotransferase, AST: aspartate aminotransferase, BMI: body mass index, CRP: C-reactive protein, DBP: diastolic blood pressure, FT3: free triiodothyronine, FT4: free thyroxine, GGT: gamma-glutamyl transpeptidase, HDL-c: high-density lipoprotein cholesterol, LDL-c: low-density lipoprotein cholesterol, MetS: metabolic syndrome, SBP: systolic blood pressure, TAG: triacylglycerol, TSH: thyroid-stimulating hormone.

**Table S3 Baseline characteristics of participants in the Optimal and Suboptimal health reference groups**

|  | **Optimal health**  **(n = 11)** | **Suboptimal health**  **(n = 15)** | **FDR** |
| --- | --- | --- | --- |
| **Number (n)** | 11 | 15 | - |
| **Age, years** | 28.36 ± 2.62 | 59.93 ± 5.54 | - |
| **Male, n(%)** | 6 (55) | 7 (47) | > 0.99 |
| **Smoker, n (%)** | 2 (18) | 2 (13) | > 0.99 |
| **Alcohol drinker, n (%)** | 4 (36) | 8 (53) | 0.80 |
| **MET-h per week, h** | 90.5 [61.4, 135.5] | 57.0 [37.6, 83.2] | 0.24 |
| **Night sleep hours** | 7.08 [7.00, 7.46] | 7.00 [7.00, 7.25] | 0.51 |
| **BMI, kg/m2** | 21.4 ± 1.65 | 26.4 ± 2.94 | - |
| **Waist circumference, cm** | 71.6 ± 6.74 | 90.4 ± 9.32 | < 0.001 |
| **Body fat percentage, %** | 21.6 ± 6.7 | 34.4 ± 6.4 | < 0.001 |
| **Trunk fat percentage, %** | 21.6 ± 5.49 | 38 ± 6.33 | < 0.001 |
| **Lean body mass, kg** | 46.1 ± 9.52 | 46.6 ± 9.26 | > 0.99 |
| **SBP, mm Hg** | 109 ± 9.82 | 119 ± 7.75 | 0.048 |
| **DBP, mm Hg** | 73.5 ± 7.16 | 80.2 ± 6.93 | 0.066 |
| **Fasting glucose, mmol/L** | 5.33 ± 0.347 | 5.91 ± 0.518 | 0.008 |
| **HbA1c, %** | 5.69 ± 0.164 | 6.17 ± 0.388 | < 0.001 |
| **Fasting insulin, IU/L** | 6.47 ± 2.76 | 8.35 ± 4.01 | 0.31 |
| **Total cholesterol, mmol/L** | 4.13 ± 0.339 | 4.6 ± 0.838 | 0.15 |
| **HDL-c, mmol/L** | 1.49 ± 0.237 | 1.39 ± 0.328 | 0.54 |
| **LDL-c, mmol/L** | 2.55 [2.37, 2.67] | 3.03 [2.46, 3.44] | 0.14 |
| **TAG, mmol/L** | 0.78 [0.65, 0.855] | 1 [0.915, 1.3] | 0.048 |
| **FT3, pmol/L** | 4.67 ± 0.634 | 4.88 ± 0.468 | 0.54 |
| **FT4, pmol/L** | 17 ± 2.39 | 15.6 ± 1.68 | 0.25 |
| **TSH, IU/L** | 1.72 [1.52, 2.56] | 2.00 [1.60, 3.58] | 0.57 |
| **AST, U/L** | 19 [17, 21] | 21 [18, 28.5] | 0.39 |
| **ALT, U/L** | 13 [10, 15] | 14 [11, 21] | 0.54 |
| **GGT, U/L** | 14 [8.5, 16] | 13 [11, 21.5] | 0.54 |
| **eGFR, mL/min per 1.73 m2** | 105 ± 18.4 | 102 ± 15.6 | 0.85 |
| **Creatinine, umol/L** | 77.9 ± 14.9 | 69.5 ± 10.1 | 0.25 |
| **CRP, mg/L** | 0.20 [0.10, 0.25] | 0.50 [0.20, 1.05] | 0.066 |

The data are presented as the mean ± standard deviation or median (1^st^ quartile, 3^rd^ quartile) for continuous variables and n (%) for categorical variables. Student’s *t*-test or the Wilcoxon signed-rank test was used to calculate the *P* values of continuous variables. Pearson’s χ2 test was used to calculate the *P* values of categorical variables. The FDR was computed using the Benjamini–Hochberg method. ALT: alanine aminotransferase, AST: aspartate aminotransferase, BMI: body mass index, CRP: C-reactive protein, DBP: diastolic blood pressure, FT3: free triiodothyronine, FT4: free thyroxine, GGT: gamma-glutamyl transpeptidase, HDL-c: high-density lipoprotein cholesterol, LDL-c: low-density lipoprotein cholesterol, MET-h: metabolic equivalent task hours, MetS: metabolic syndrome, SBP: systolic blood pressure, TAG: triacylglycerol, THS: thyroid-stimulating hormone.

**Table S4 Values of metabolic treats in different subgroups with high and low scores in HRSs and HPSs**

| Axis | Metabolic traits | LHPS-LHRS (n = 31) | HHPS-LHRS (n = 24) | LHPS-HHRS (n = 24) | HHPS-HHRS (n = 32) | FDR |
| --- | --- | --- | --- | --- | --- | --- |
| Overall | Body fat (%) | 33.4 ± 5.62 | 25.3 ± 7.89 | 31.0 ± 8.51 | 23.5 ± 6.76 | < 0.001 |
|  | SBP (mmHg) | 128 ± 13. | 113 ± 10.9 | 127 ± 13.9 | 116 ± 13.2 | 0.033 |
|  | DBP (mmHg) | 87.1 ± 11.0 | 76.0 ± 8.37 | 86.5 ± 11.04 | 77.7 ± 9.42 | 0.032 |
|  | Glucose (mmol/L) | 5.82 ± 0.52 | 5.55 ± 0.60 | 5.94 ± 0.5 | 5.33 ± 0.35 | 0.014 |
|  | Insulin (uIU/mL) | 8.70 [5.40, 13.8] | 6.50 [3.48, 9.00] | 8 [5.75, 11.22] | 6.85 [4.85, 8.72] | 0.15 |
|  | C-peptide (ng/mL) | 1.26 [0.95, 1.53] | 0.72 [0.63, 0.94] | 1.06 [0.78, 1.21] | 0.87 [0.59, 0.96] | < 0.001 |
|  | HbA1c (%) | 6.00 [5.75, 6.40] | 5.70 [5.57, 5.90] | 6 [5.8, 6.3] | 5.7 [5.57, 5.9] | < 0.001 |
|  | HOMA-IR | 2.27 [1.27, 3.42] | 1.53 [0.67, 2.33] | 2.08 [1.54, 3.23] | 1.6 [1.14, 2.11] | 0.056 |
|  | Matsuda index | 4.90 [3.38, 6.53] | 6.93 [5.87, 13.91] | 5.39 [3.66, 7.33] | 8.28 [5.4, 9.8] | 0.002 |
|  | HDL-c (mmol/L) | 1.26 ± 0.24 | 1.49 ± 0.35 | 1.36 ± 0.43 | 1.45 ± 0.3 | 0.092 |
|  | LDL-c (mmol/L) | 3.19 [2.9, 3.67] | 2.79 [2.21, 3.24] | 3.26 [2.72, 3.67] | 2.55 [2.25, 2.93] | 0.002 |
|  | TCH (mmol/L) | 4.82 [4.46, 5.20] | 4.32 [3.99, 5.16] | 4.89 [4.47, 5.64] | 4.2 [3.95, 4.57] | 0.003 |
|  | TAG (mmol/L) | 1.50 [1.23, 1.77] | 0.80 [0.66, 1.01] | 1.10 [0.77, 1.67] | 0.91 [0.79, 1.24] | < 0.001 |
|  | ALT (U/l) | 18.0 [12.5, 23.0] | 11.5 [7.00, 21.3] | 19.0 [9.75, 22.0] | 13.0 [10.0, 17.3] | 0.073 |
|  | AST (U/l) | 26.0 [21.0, 32.0] | 22.0 [19.8, 25.3] | 21.5 [19.8, 26.0] | 20.0 [18.0, 23.0] | 0.011 |
|  | GGT (U/l) | 22.0 [18.5, 29.5] | 14.0 [11.8, 18.3] | 16.5 [11.8, 21.3] | 15.0 [12.8, 19.3] | 0.014 |
|  | Creatin (mg/dL) | 68.9 ± 11.4 | 75.0 ± 15.1 | 75.5 ± 12.9 | 76.5 ± 14.9 | 0.051 |
|  | Uric acid | 3682 ± 415 | 3521 ± 352 | 3717 ± 410 | 3707 ± 407 | 0.51 |
|  | Adiponectin (ug/mL) | 4.48 [3.20, 6.91] | 7.82 [4.65, 9.96] | 6.03 [5.23, 9.45] | 6.07 [4.56, 9.52] | 0.025 |
|  | Leptin (ng/mL) | 4.18 [2.31, 7.15] | 2.20 [0.59, 3.09] | 2.39 [1.24, 4.35] | 0.77 [0.39, 2.28] | < 0.001 |
|  | CRP (mg/L) | 0.60 [0.20, 1.45] | 0.20 [0.10, 1.02] | 0.50 [0.30, 1.20] | 0.20 [0.10, 0.50] | 0.014 |
|  | SAA-1 (ug/mL) | 1.19 [0.61, 1.85] | 0.74 [0.47, 1.31] | 1.17 [0.48, 1.62] | 0.61 [0.34, 1.33] | 0.22 |
|  | AAA (umol/L) | 268 ± 28.4 | 255 ± 39.2 | 253 ± 24.3 | 240 ± 27.9 | 0.002 |
|  | BCAA (umol/L) | 2446 [2067, 2701] | 2378 [2030, 2760] | 2306 [2181, 2664] | 2216 [2020, 2631] | 0.65 |
|  | Metabolic traits | LHPS-LHRS (n = 33) | HHPS-LHRS (n = 22) | LHPS-HHRS (n = 22) | HHPS-HHRS (n = 34) | FDR |
| Axis-glu | Body fat (%) | 31.8 ± 7.87 | 28.6 ± 8.50 | 29.3 ± 6.69 | 23.9 ± 7.55 | 0.001 |
|  | SBP (mmHg) | 127 ± 15.6 | 117 ± 10.8 | 121 ± 13.8 | 117 ± 14.3 | 0.02 |
|  | DBP (mmHg) | 87.2 ± 11.5 | 78.8 ± 10.1 | 81.9 ± 11.4 | 78.6 ± 9.54 | 0.011 |
|  | Glucose (mmol/L) | 5.86 ± 0.40 | 5.38 ± 0.60 | 6.02 ± 0.55 | 5.38 ± 0.38 | 0.021 |
|  | Insulin (uIU/mL) | 9.00 [6.50, 15.2] | 7.05 [4.80, 11.4] | 7.35 [5.53, 9.98] | 5.65 [3.92, 7.62] | 0.014 |
|  | C-peptide (ng/mL) | 1.08 [0.92, 1.51] | 0.72 [0.59, 1.14] | 1.04 [0.77, 1.15] | 0.77 [0.60, 0.95] | < 0.001 |
|  | HbA1c (%) | 6.00 [5.90, 6.40] | 5.60 [5.40, 5.70] | 6.10 [5.80, 6.30] | 5.70 [5.50, 5.90] | < 0.001 |
|  | HOMA-IR | 2.35 [1.61, 3.95] | 1.66 [1.18, 2.73] | 1.94 [1.51, 3.04] | 1.33 [0.88, 1.79] | 0.004 |
|  | Matsuda index | 4.13 [3.03, 6.11] | 6.67 [4.31, 8.72] | 6.25 [4.51, 7.58] | 8.71 [6.54, 13.39] | < 0.001 |
|  | HDL-c (mmol/L) | 1.27 ± 0.31 | 1.36 ± 0.27 | 1.43 ± 0.35 | 1.48 ± 0.37 | 0.014 |
|  | LDL-c (mmol/L) | 3.03 [2.58, 3.41] | 2.71 [2.27, 3.52] | 3.23 [2.97, 3.48] | 2.62 [2.38, 3.12] | 0.067 |
|  | TCH (mmol/L) | 4.73 [4.17, 5.14] | 4.44 [4, 5.48] | 4.73 [4.54, 5.51] | 4.26 [4.1, 4.64] | 0.085 |
|  | TAG (mmol/L) | 1.50 [0.98, 1.81] | 1.23 [0.88, 1.59] | 1.07 [0.74, 1.40] | 0.85 [0.77, 1.01] | 0.002 |
|  | ALT (U/l) | 20.0 [12.0, 22.0] | 12.0 [10.3, 15.5] | 14.0 [9.25, 19.0] | 14.5 [10.0, 22.0] | 0.10 |
|  | AST (U/l) | 26.0 [20.0, 31.0] | 20.5 [18.0, 23.0] | 22.0 [20.3, 26.8] | 20.0 [19.0, 25.5] | 0.11 |
|  | GGT (U/l) | 22.0 [16.0, 33.0] | 13.5 [10.3, 18.8] | 15.5 [11.0, 20.0] | 17.0 [14.0, 21.5] | 0.003 |
|  | Creatin (mg/dL) | 72.5 ± 14.9 | 73.6 ± 13.2 | 70.1 ± 12.7 | 77.8 ± 13.4 | 0.22 |
|  | Uric acid | 3528 ± 379 | 3762 ± 389 | 3679 ± 478 | 3716 ± 355 | 0.12 |
|  | Adiponectin (ug/mL) | 5.25 [3.96, 7.05] | 5.40 [2.83, 7.29] | 6.51 [4.71, 9.55] | 8.30 [5.30, 11.2] | 0.005 |
|  | Leptin (ng/mL) | 4.04 [1.94, 7.20] | 1.60 [0.82, 5.36] | 2.13 [1.19, 4.12] | 1.13 [0.33, 2.91] | 0.007 |
|  | CRP (mg/L) | 0.70 [0.30, 1.60] | 0.50 [0.10, 0.88] | 0.40 [0.30, 1.05] | 0.20 [0.10, 0.48] | 0.014 |
|  | SAA-1 (ug/mL) | 1.30 [0.55, 2.26] | 0.91 [0.62, 1.71] | 0.79 [0.44, 1.49] | 0.59 [0.27, 1.18] | 0.035 |
|  | AAA (umol/L) | 263 ± 33.5 | 257 ± 32.7 | 250 ± 21.1 | 243 ± 32.2 | 0.014 |
|  | BCAA (umol/L) | 2279 [1982, 2713] | 2527 [2162, 2713] | 2306 [2177, 2588] | 2222 [2050, 2552] | 0.34 |
|  | Metabolic traits | LHPS-LHRS (n = 33) | HHPS-LHRS (n = 21) | LHPS-HHRS (n = 22) | HHPS-HHRS (n = 35) | FDR |
| Axis-lip | Body fat (%) | 33.5 ± 6.23 | 22.9 ± 6.98 | 33.3 ± 6.41 | 23.4 ± 6.48 | < 0.001 |
|  | SBP (mmHg) | 128 ± 15.0 | 116 ± 13.8 | 124 ± 11.7 | 115 ± 12.6 | 0.005 |
|  | DBP (mmHg) | 87.8 ± 11.4 | 76.0 ± 10.1 | 84.9 ± 10.6 | 77.9 ± 8.46 | 0.009 |
|  | Glucose (mmol/L) | 5.90 ± 0.44 | 5.54 ± 0.60 | 5.76 ± 0.48 | 5.40 ± 0.52 | 0.002 |
|  | Insulin (uIU/mL) | 10.9 [7.70, 15.8] | 6.50 [3.90, 9.60] | 6.40 [5.43, 9.98] | 5.40 [3.55, 7.60] | 0.001 |
|  | C-peptide (ng/mL) | 1.26 [1.01, 1.50] | 0.90 [0.65, 1.07] | 0.93 [0.78, 1.19] | 0.72 [0.59, 0.90] | < 0.001 |
|  | HbA1c (%) | 6.00 [5.70, 6.30] | 5.70 [5.60, 5.90] | 6.05 [5.80, 6.3] | 5.80 [5.60, 5.90] | 0.006 |
|  | HOMA-IR | 3.03 [2.08, 3.99] | 1.59 [1.13, 2.44] | 1.57 [1.36, 2.78] | 1.43 [0.85, 1.83] | < 0.001 |
|  | Matsuda index | 3.74 [3.18, 5.34] | 6.93 [5.89, 11.2] | 5.84 [4.32, 7.7] | 8.32 [6.32, 13.3] | < 0.001 |
|  | HDL-c (mmol/L) | 1.25 ± 0.30 | 1.46 ± 0.35 | 1.32 ± 0.34 | 1.50 ± 0.32 | 0.014 |
|  | LDL-c (mmol/L) | 3.31 [2.94, 3.85] | 2.40 [2.13, 3.01] | 2.93 [2.49, 3.33] | 2.94 [2.47, 3.27] | 0.004 |
|  | TCH (mmol/L) | 4.91 [4.50, 5.38] | 4.17 [3.97, 4.37] | 4.52 [4.16, 5.34] | 4.51 [4.13, 4.88] | 0.012 |
|  | TAG (mmol/L) | 1.52 [1.21, 1.81] | 0.89 [0.66, 1.33] | 1.22 [0.83, 1.56] | 0.87 [0.76, 0.98] | < 0.001 |
|  | ALT (U/l) | 18.0 [13.0, 25.0] | 12.0 [7.00, 22.0] | 13.00 [10.3, 20.8] | 14.0 [10.0, 18.0] | 0.081 |
|  | AST (U/l) | 25.0 [20.0, 32.0] | 22.0 [19.0, 24.0] | 21.5 [19.0, 25.3] | 21.0 [19.0, 26.5] | 0.44 |
|  | GGT (U/l) | 22.0 [14.0, 30.0] | 15.0 [11.0, 19.0] | 16.0 [11.0, 21.8] | 15.0 [13.0, 20.0] | 0.032 |
|  | Creatin (mg/dL) | 70.2 ± 11.9 | 78.0 ± 15.6 | 71.3 ± 13.0 | 76.4 ± 14.2 | 0.19 |
|  | Uric acid | 3604 ± 386 | 3545 ± 278 | 3674 ± 379 | 3779 ± 468 | 0.06 |
|  | Adiponectin (ug/mL) | 4.49 [3.29, 6.27] | 6.76 [3.17, 8.85] | 6.11 [5.05, 8.12] | 7.54 [5.41, 11.07] | 0.005 |
|  | Leptin (ng/mL) | 4.81 [2.43, 7.56] | 1.52 [0.29, 2.8] | 2.58 [1.51, 4.25] | 0.81 [0.41, 2.32] | < 0.001 |
|  | CRP (mg/L) | 0.70 [0.30, 1.20] | 0.20 [0.10, 1.10] | 0.40 [0.20, 1.32] | 0.20 [0.10, 0.50] | 0.014 |
|  | SAA-1 (ug/mL) | 1.03 [0.55, 1.62] | 0.61 [0.44, 1.51] | 0.98 [0.42, 1.65] | 0.68 [0.41, 1.37] | 0.45 |
|  | AAA (umol/L) | 266 ± 27.5 | 259 ± 34.7 | 248 ± 24.2 | 243 ± 33.7 | 0.004 |
|  | BCAA (umol/L) | 2446 [2170, 2707] | 2616 [2075, 2889] | 2278 [2152, 2496] | 2239 [2004, 2614] | 0.36 |
|  | Metabolic traits | LHPS-LHRS (n = 32) | HHPS-LHRS (n = 23) | LHPS-HHRS (n = 23) | HHPS-HHRS (n = 33) | FDR |
| Axis-aa | Body fat (%) | 32.2 ± 5.74 | 25.7 ± 8.28 | 29.7 ± 8.54 | 25.2 ± 8.37 | 0.009 |
|  | SBP (mmHg) | 126 ± 12.6 | 113 ± 9.39 | 127 ± 14.4 | 116. ± 15.7 | 0.13 |
|  | DBP (mmHg) | 86.2 ± 10.3 | 75.2 ± 6.93 | 86.3 ± 10.7 | 79.2 ± 11.8 | 0.15 |
|  | Glucose (mmol/L) | 5.76 ± 0.56 | 5.64 ± 0.45 | 5.90 ± 0.62 | 5.36 ± 0.40 | 0.025 |
|  | Insulin (uIU/mL) | 6.80 [4.70, 12.7] | 8.80 [4.15, 11.2] | 7.40 [5.55, 8.85] | 6.50 [4.10, 7.80] | 0.68 |
|  | C-peptide (ng/mL) | 1.13 [0.89, 1.41] | 0.82 [0.64, 1.00] | 0.93 [0.72, 1.16] | 0.88 [0.60, 1.05] | 0.006 |
|  | HbA1c (%) | 6.00 [5.70, 6.30] | 5.70 [5.60, 5.90] | 6.00 [5.75, 6.15] | 5.80 [5.60, 5.90] | 0.016 |
|  | HOMA-IR | 1.71 [1.21, 3.39] | 2.30 [1.09, 2.73] | 1.86 [1.38, 2.42] | 1.50 [0.97, 2.07] | 0.48 |
|  | Matsuda index | 5.40 [3.48, 7.67] | 6.30 [4.94, 9.15] | 6.30 [4.35, 7.71] | 8.26 [4.98, 11.2] | 0.14 |
|  | HDL-c (mmol/L) | 1.25 ± 0.24 | 1.43 ± 0.37 | 1.39 ± 0.42 | 1.48 ± 0.30 | 0.018 |
|  | LDL-c (mmol/L) | 3.16 [2.82, 3.55] | 2.69 [2.23, 3.22] | 3.24 [2.90, 3.81] | 2.55 [2.24, 2.94] | 0.003 |
|  | TCH (mmol/L) | 4.78 [4.43, 5.18] | 4.28 [3.96, 5.06] | 4.95 [4.51, 5.69] | 4.21 [3.93, 4.58] | 0.004 |
|  | TAG (mmol/L) | 1.35 [1.01, 1.75] | 0.87 [0.68, 1.10] | 1.28 [0.78, 1.61] | 0.90 [0.69, 1.31] | 0.002 |
|  | ALT (U/l) | 20.5 [12.8, 27.8] | 13.0 [11.0, 20.5] | 14.0 [9.5, 20.0] | 13.0 [10.0, 18.0] | 0.054 |
|  | AST (U/l) | 27.5 [20.0, 32.3] | 21.0 [19.0, 24.5] | 22.0 [20.0, 26.0] | 20.0 [18.0, 24.0] | 0.014 |
|  | GGT (U/l) | 20.5 [15.3, 27.0] | 15.0 [11.5, 20.5] | 17.0 [12.0, 21.0] | 15.0 [14.0, 19.0] | 0.19 |
|  | Creatin (mg/dL) | 71.5 ± 11.9 | 75.4 ± 15.2 | 75.7 ± 13.0 | 73.8 ± 15.3 | 0.54 |
|  | Uric acid | 3693 ± 370 | 3618 ± 440 | 3690 ± 392 | 3644 ± 421 | 0.76 |
|  | Adiponectin (ug/mL) | 4.87 [3.27, 7.10] | 7.15 [4.06, 10.1] | 5.95 [5.42, 9.98] | 7.08 [3.98, 9.30] | 0.083 |
|  | Leptin (ng/mL) | 3.26 [2.21, 5.90] | 2.41 [0.56, 3.87] | 1.40 [0.92, 3.67] | 1.21 [0.38, 3.84] | 0.025 |
|  | CRP (mg/L) | 0.65 [0.20, 1.70] | 0.40 [0.10, 1.05] | 0.40 [0.30, 0.60] | 0.20 [0.10, 0.50] | 0.027 |
|  | SAA-1 (ug/mL) | 1.02 [0.66, 1.61] | 0.80 [0.52, 1.37] | 1.55 [0.52, 1.71] | 0.55 [0.34, 1.33] | 0.15 |
|  | AAA (umol/L) | 269 ± 27.7 | 257 ± 35.9 | 248 ± 25.9 | 240 ± 29.3 | < 0.001 |
|  | BCAA (umol/L) | 2358 [2144, 2695] | 2447 [2015, 3016] | 2301 [2153, 2406] | 2243 [2033, 2650] | 0.68 |

Data are shown as mean ± standard error and medians [1^st^ quartile, 3^rd^ quartile] for normal and non-normal distribution values. Benjamini–Hochberg FDR values were calculated from tests conducted using ANOVA or the Kruskal-Wallis test. aa: amino acid, AAA: aromatic amino acids, ALT: alanine aminotransferase, AST: aspartate aminotransferase, BCAA: branch chain amino acid, CRP: C-reactive protein, DPB: diastolic blood pressure, GGT: gamma-glutamyl transpeptidase, glu: glucose, HDL-c: high-density lipoprotein cholesterol, HHPS: high health phenotype score, HHRS: high homeostatic resilience score, LDL-c: low-density lipoprotein cholesterol, LHRS: low homeostatic resilience score, lip: lipid, LHPS: low health phenotype score, SAA-1: serum amyloid protein-1, SBP: systolic blood pressure, TAG: triacylglycerol, TCH: total cholesterol.

**Table S5 Multi-timepoint biomarkers at post-MMTT used to compute the HRS (total n = 141, significantly changed n = 101)**

| **Feature** | **Axis** | **FDR_time_** | **Feature** | **Axis** | **FDR_time_** |
| --- | --- | --- | --- | --- | --- |
| 1,5-anhydroglucitol | Glucose | < 0.001 | FMN | Amino acid | 0.278 |
| 3-Methyl-2-oxovaleric acid | Glucose | < 0.001 | Glutamate | Amino acid | < 0.001 |
| 3-phosphoglycerate | Glucose | 0.33 | Glutamine | Amino acid | 0.142 |
| 4-Methyl-2-oxovaleric acid | Glucose | 0.114 | Histidine | Amino acid | 0.002 |
| C-peptide | Glucose | < 0.001 | Homocysteine | Amino acid | 0.878 |
| Fructose | Glucose | 0.595 | Isoleucine | Amino acid | < 0.001 |
| GIP | Glucose | < 0.001 | Leucine | Amino acid | 0.001 |
| GLP1 | Glucose | < 0.001 | Lysine | Amino acid | < 0.001 |
| Ghrelin | Glucose | < 0.001 | Methionine | Amino acid | < 0.001 |
| Glucagon | Glucose | < 0.001 | Ornithine | Amino acid | < 0.001 |
| Glucose | Glucose | < 0.001 | Pantothenic acid | Amino acid | < 0.001 |
| Glyceraldehyde-3-phosphate | Glucose | < 0.001 | Phenylalanine | Amino acid | < 0.001 |
| Glycerol-3-phosphate | Glucose | < 0.001 | Proline | Amino acid | < 0.001 |
| Insulin | Glucose | < 0.001 | Serine | Amino acid | < 0.001 |
| Isoleucine | Glucose | < 0.001 | Taurine | Amino acid | < 0.001 |
| L-Lactate | Glucose | < 0.001 | Threonine | Amino acid | < 0.001 |
| Leucine | Glucose | 0.001 | Tryptophan | Amino acid | < 0.001 |
| Pyruvate | Glucose | < 0.001 | Tyrosine | Amino acid | < 0.001 |
| UDP-glucose | Glucose | 0.114 | Valine | Amino acid | < 0.001 |
| Valine | Glucose | < 0.001 | 17-OH Progesterone | Others | 0.704 |
| 3-Hydroxybutanoic acid | Lipid | < 0.001 | 2-Hydroxybenzoic acid | Others | 0.002 |
| Aconitate | Lipid | < 0.001 | 3-Methylhistidine | Others | < 0.001 |
| Adiponectin | Lipid | 0.249 | 5-Hydroxy-L-tryptophan | Others | < 0.001 |
| Alpha ketoglutarate | Lipid | 0.406 | ADP | Others | 0.113 |
| Acylcarnitine | Lipid | 0.013 | ALT | Others | < 0.001 |
| Acylcarnitine C10 | Lipid | < 0.001 | AMP | Others | 0.113 |
| Acylcarnitine C10:1 | Lipid | < 0.001 | AST | Others | 0.225 |
| Acylcarnitine C10:OH | Lipid | < 0.001 | ATP | Others | 0.457 |
| Acylcarnitine C12 | Lipid | < 0.001 | CEA | Others | 0.568 |
| Acylcarnitine C12:1 | Lipid | < 0.001 | CRP | Others | 0.159 |
| Acylcarnitine C12OH | Lipid | < 0.001 | Catechin | Others | 0.913 |
| Acylcarnitine C14 | Lipid | < 0.001 | Citrulline | Others | < 0.001 |
| Acylcarnitine C14:1 | Lipid | < 0.001 | Cortisol | Others | < 0.001 |
| Acylcarnitine C14:2 | Lipid | < 0.001 | Dopamine | Others | < 0.001 |
| Acylcarnitine C14OH | Lipid | < 0.001 | E-selectin | Others | < 0.001 |
| Acylcarnitine C16 | Lipid | 0.292 | FT3 | Others | < 0.001 |
| Acylcarnitine C16:1 | Lipid | < 0.001 | FT4 | Others | < 0.001 |
| Acylcarnitine C16:1OH | Lipid | < 0.001 | Flavin adenine dinucleotide | Others | 0.076 |
| Acylcarnitine C16OH | Lipid | < 0.001 | GALA | Others | < 0.001 |
| Acylcarnitine C18 | Lipid | < 0.001 | GGT | Others | 0.005 |
| Acylcarnitine C18:1 | Lipid | < 0.001 | GOLA | Others | 0.724 |
| Acylcarnitine C18:2OH | Lipid | < 0.001 | Glucosepane | Others | 0.006 |
| Acylcarnitine C18OH | Lipid | 0.188 | Hippuric acid | Others | < 0.001 |
| Acylcarnitine C3 | Lipid | < 0.001 | Hypoxanthine | Others | < 0.001 |
| Acylcarnitine C4DC | Lipid | < 0.001 | ICAM-1 | Others | < 0.001 |
| Acylcarnitine C5 | Lipid | < 0.001 | IFN-γ | Others | 0.086 |
| Acylcarnitine C5OH | Lipid | < 0.001 | IL10 | Others | 0.58 |
| Acylcarnitine C6 | Lipid | < 0.001 | IL18 | Others | 0.55 |
| Acylcarnitine C6DC | Lipid | 0.406 | IL1b | Others | 0.71 |
| Acylcarnitine C8 | Lipid | < 0.001 | IL6 | Others | < 0.001 |
| Acylcarnitine C8:1 | Lipid | < 0.001 | IL8 | Others | 0.086 |
| Citrate | Lipid | < 0.001 | Inosine-5-monophosphate | Others | 0.402 |
| Deoxycholic acid | Lipid | < 0.001 | Inositol | Others | 0.017 |
| FFA | Lipid | < 0.001 | MODIC | Others | < 0.001 |
| HDL-c | Lipid | 0.026 | Metanephrine | Others | 0.01 |
| Isocitrate | Lipid | < 0.001 | N6-acetyl lysine | Others | < 0.001 |
| L-Malate | Lipid | < 0.001 | N6-formyl | Others | 0.059 |
| LDL-c | Lipid | < 0.001 | N6-glycerinyl | Others | 0.387 |
| Leptin | Lipid | 0.067 | N6-threonyl | Others | < 0.001 |
| Succinate | Lipid | 0.474 | Na-methyl-L-ornithine monohydrochloride | Others | < 0.001 |
| TAG | Lipid | < 0.001 | Normetanephrine | Others | 0.067 |
| Total cholesterol | Lipid | < 0.001 | P-selectin | Others | < 0.001 |
| 1-Methylhistidine | Amino acid | < 0.001 | Progesterone | Others | 0.103 |
| 3-Aminoisobutyric acid | Amino acid | < 0.001 | Quinic acid | Others | 0.012 |
| Arginine | Amino acid | < 0.001 | Ribose | Others | < 0.001 |
| Asparagine | Amino acid | < 0.001 | SAA1 | Others | < 0.001 |
| Aspartate | Amino acid | < 0.001 | TNF-α | Others | 0.119 |
| Beta-alanine | Amino acid | < 0.001 | TSH | Others | < 0.001 |
| Cobalamin | Amino acid | 0.119 | Uric acid | Others | 0.393 |
| Creatinine | Amino acid | < 0.001 | VCAM-1 | Others | < 0.001 |
| Cysteine | Amino acid | 0.812 | VEGFA | Others | 0.225 |
| Cystine | Amino acid | < 0.001 | Vanillylmandelic acid | Others | 0.913 |

The total number of features was not equivalent to the summation of those on the three axes and others, as some features were duplicated in the three axes. Post-MMTT changes were tested using a mixed-effects model with time as the fixed effect and participants as the random effect; both variables were treated as factor variables. FDR_time_ denotes multiple tests corrected by the Benjamini–Hochberg false discovery rate for time. ADP: adenosine diphosphate, ALT: alanine aminotransferase, AMP: adenosine monophosphate, AST: aspartate aminotransferase, ATP: adenosine triphosphate, CEA: N7-carboxyethylarginine, CRP: C-reactive protein, DBP: diastolic blood pressure, FFA: free fatty acid, FT3: free triiodothyronine, FT4: free thyroxine, GALA: glycolic acid lysine amide, GGT: gamma-glutamyl transpeptidase, GIP: gastric inhibitory polypeptide, GLP-1: glucagon-like peptide-1, GOLA: glyoxal lysine amide, HDL-c: high-density lipoprotein cholesterol, ICAM-1: intercellular cell adhesion molecule-1, IFN-g: interferon gamma, IL: interleukin, LDL-c: low-density lipoprotein cholesterol, MODIC: methylglyoxal imidazolinone, SAA-1: serum amyloid protein-1, SBP: systolic blood pressure, TAG: triacylglycerol, TNF-α: tumour necrosis factor-α, TSH: thyroid-stimulating hormone, VCAM-1: vascular cell adhesion molecule-1, VEGFA: vascular endothelial growth factor receptor.

**Table S6 Summary of tested SNPs involving in postprandial glucose, TAG, and insulin responses**

| **Chr** | **Pos (bp)** | **rs ID** | **EA** | **NEA** | **Gene (nearest)** | **Frequency of EA** | **Effect** | **SE** | ***P* value** | **Phenotype** | **Challenge type** |
| --- | --- | --- | --- | --- | --- | --- | --- | --- | --- | --- | --- |
| 2 | 27730940 | rs1260326 | T | C | GCKR | 0.40 | 0.049 | 0.0078 | 5.93E-12 | 2hGlu | OGTT |
| 2 | 165558252 | rs12692738 | T | C | COBLL1 | 0.76 | 0.049 | 0.0090 | 2.72E-08 | 2hGlu | OGTT |
| 3 | 185526062 | rs7630554 | A | G | IGF2BP2 | 0.70 | -0.056 | 0.0083 | 1.56E-11 | 2hGlu | OGTT |
| 7 | 44255643 | rs878521 | A | G | CAMK2B | 0.24 | 0.099 | 0.0094 | 1.25E-28 | 2hGlu | OGTT |
| 7 | 44231778 | rs2971669 | T | C | GCK | 0.21 | 0.10 | 0.0099 | 3.82E-28 | 2hGlu | OGTT |
| 8 | 9183596 | rs4841132 | A | G | LOC157273 | 0.11 | -0.071 | 0.013 | 3.13E-09 | 2hGlu | OGTT |
| 10 | 114758349 | rs7903146 | T | C | TCF7L2 | 0.26 | 0.085 | 0.0087 | 2.79E-26 | 2hGlu | OGTT |
| 11 | 17415190 | rs4148646 | C | G | ABCC8 | 0.40 | 0.040 | 0.0078 | 4.39E-08 | 2hGlu | OGTT |
| 14 | 38842759 | rs112824462 | A | G | CLEC14A | 0.25 | -0.058 | 0.010 | 6.73E-09 | 2hGlu | OGTT |
| 15 | 62332980 | rs17271305 | A | G | VPS13C | 0.58 | -0.059 | 0.0077 | 2.88E-14 | 2hGlu | OGTT |
| 15 | 62330633 | rs11634163 | T | C | VPS13C | 0.42 | 0.058 | 0.0077 | 3.29E-14 | 2hGlu | OGTT |
| 19 | 46181392 | rs1800437 | C | G | GIPR | 0.22 | 0.10 | 0.0099 | 4.79E-26 | 2hGlu | OGTT |
| 19 | 46182304 | rs10423928 | A | T | GIPR | 0.22 | 0.10 | 0.0099 | 8.64E-26 | 2hGlu | OGTT |
| 6 | 20679709 | rs7756992 | G | A | CDKAL1 | 0.30 | -0.11 | 0.015 | 3.07E-13 | CIR | OGTT |
| 7 | 50758245 | rs933360 | A | G | GRB10 | 0.62 | -0.051 | 0.0086 | 3.14E-09 | CIR | OGTT |
| 7 | 44231216 | rs3757840 | T | G | GCK | 0.45 | -0.090 | 0.015 | 1.34E-09 | CIR | OGTT |
| 8 | 41509259 | rs12549902 | A | G | ANK1 | 0.58 | -0.060 | 0.010 | 1.01E-08 | CIR | OGTT |
| 10 | 94482076 | rs7923866 | C | T | HHEX/IDE | 0.62 | -0.12 | 0.015 | 4.16E-16 | CIR | OGTT |
| 11 | 92708710 | rs10830963 | G | C | MTNR1B | 0.69 | -0.17 | 0.016 | 6.71E-28 | CIR | OGTT |
| 15 | 62383155 | rs4502156 | T | C | C2CD4A  (NLF1/VPS13C) | 0.52 | -0.092 | 0.014 | 1.14E-10 | CIR | OGTT |
| 19 | 46172278 | rs11671664 | A | G | GIPR | 0.11 | -0.17 | 0.025 | 2.64E-11 | CIR | OGTT |
| 7 | 68904887 | rs10243693 | A | G | - | 0.36 | 0.28 | 0.051 | 3.50E-08 | TAG AUC | high-fat meal |
| 11 | 116648917 | rs964184 | C | G | ZPR1 | 0.87 | -0.41 | 0.075 | 3.82E-08 | TAG AUC^*^ | high-fat meal |

* Adjusted for fasting TAG levels. Chr: chromosome, CIS: corrected insulin response, EA: effect allele, NEA: noneffect allele, OGTT: oral glucose tolerance test, Pos: position on chromosome.


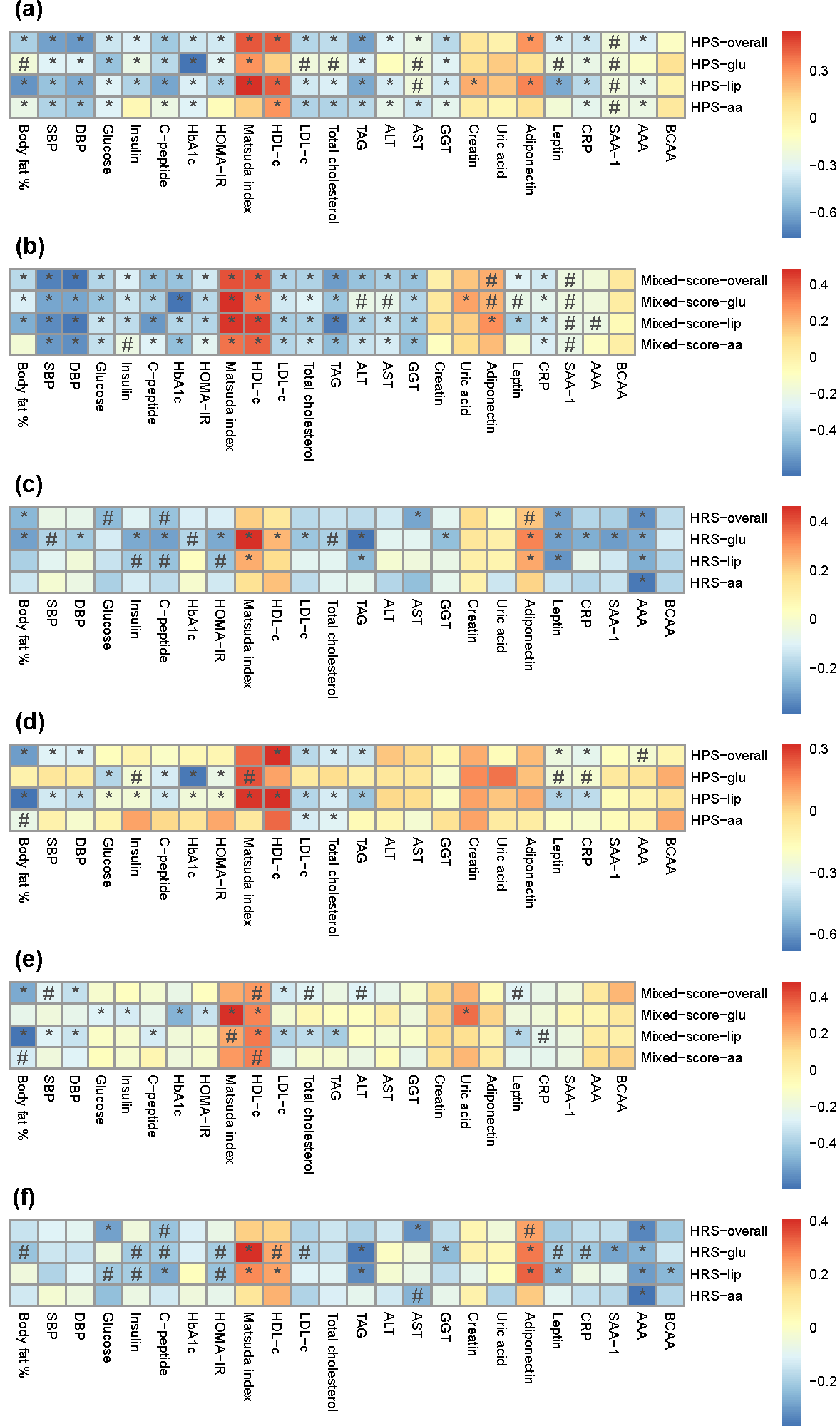


**Fig. S1 Spearman’s correlation coefficients for 24 metabolic traits with (a) HPS (n = 85), (b) the mixed-score (n = 85) and (c) HRS (n = 100), and also partial Spearman’s correlation coefficients for metabolic traits with (d) HPS (n = 85), (e) the mixed-score (n = 85) and (f) the HRS (n = 100), adjusted for age, sex, and BMI.** For the HPS, the participants in the two reference groups were excluded from the analyses. For the HRS, the participants in the optimal reference group were excluded from the analyses. The FDR was computed using the Benjamini–Hochberg method: ^#^ < 0.1, * < 0.05, ** < 0.01, *** < 0.001. AAA: aromatic amino acid, ALT: alanine aminotransferase, AST: aspartate aminotransferase, BCAA: branch chain amino acid, CRP: C-reactive protein, DBP: diastolic blood pressure, GGT: gamma-glutamyl transpeptidase, GIP: gastric inhibitory polypeptide, GLP-1: glucagon-like peptide-1, HDL-c: high-density lipoprotein cholesterol, HPS: Health Phenotype Score, HRS: homeostatic resilience score, LDL-c: low-density lipoprotein cholesterol, SAA-1: serum amyloid protein-1, SBP: systolic blood pressure, Spearman’s *r*: Spearman’s correlation coefficient, TAG: triacylglycerol.


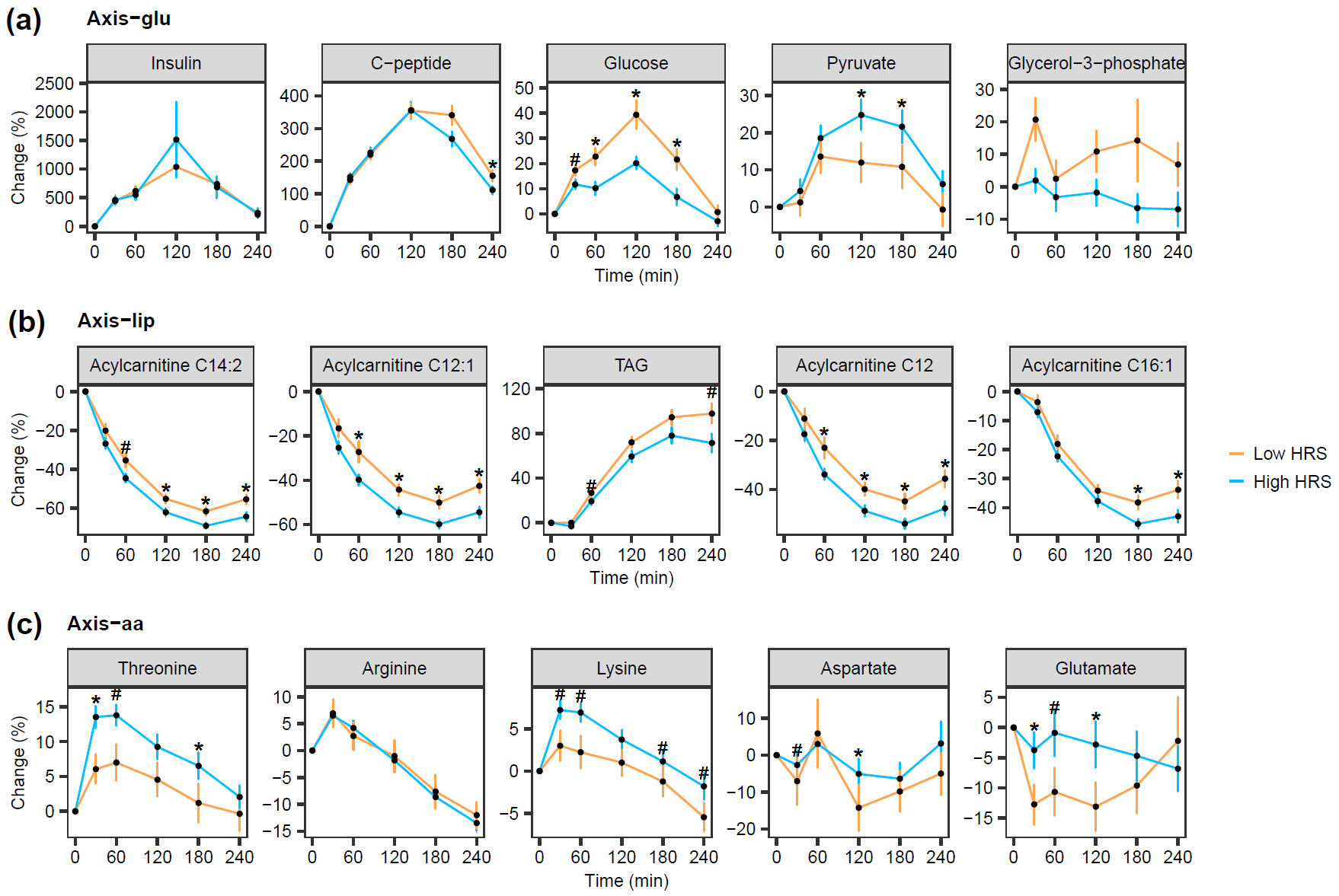


**Fig. S2 Top five features related to postprandial changes among participants with high (≥ median) or low (< median) HRSs on the three axes: (a) axis-glu, (b) axis-lip and (c) axis-aa.** The data are shown as the mean ± standard error (error bar) in line plots as a function of percentage change over time. Student’s *t*-test or Wilcoxon signed-ranks test was used to calculate *P* values, and significance levels are presented as * < 0.05, # < 0.1. aa: amino acid, glu: glucose, HRS: homeostatic resilience score, lip: lipid, TAG: triacylglycerol.


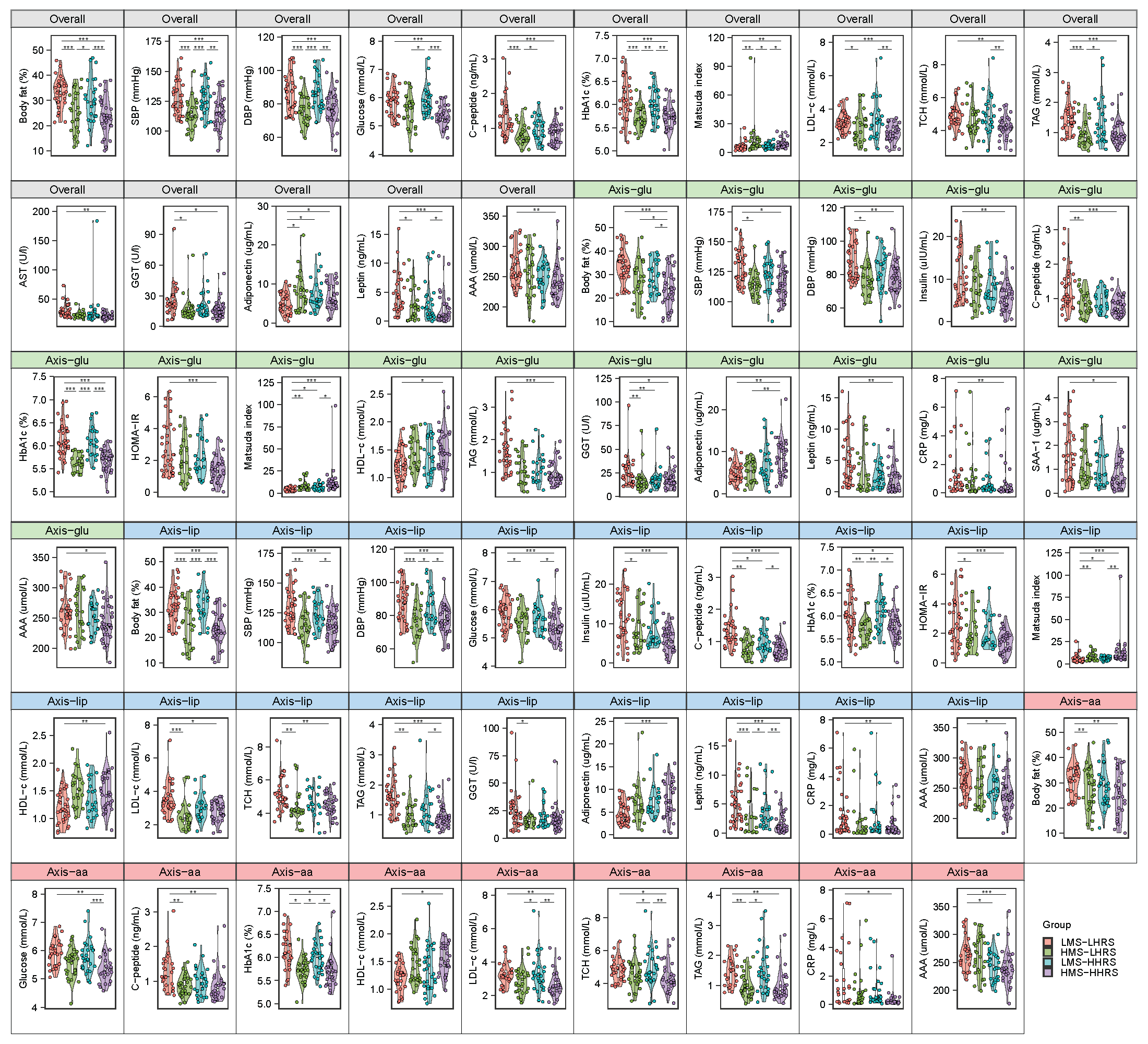


**Fig. S3 Violin plots comparing single metabolic traits among participants with low-mixed-score + low-HRS, high-mixed-score + low-HRS, low-mixed-score + high-HRS, high-mixed-score + high-HRS (n = 30, 25, 25, and 31 for overall; 32, 23, 23, and 33 for axis-glu; 33, 21, 22, and 35 for axis-lip; 26, 29, 29, and 27 for axis-aa, respectively).** Features with FDR-corrected values < 0.05, tested by ANOVA or the Kruskal-Wallis test, were displayed. Post-hoc analyses were conducted using Student's *t*-tests or pairwise Wilcoxon signed-rank tests between each pair of groups. FDR was calculated using the Benjamini–Hochberg method for multiple testing correction defined as: *** < 0.001, ** < 0.01, and * < 0.05. Boxes are shown as medians between the 1^st^ quartile and 3^rd^ quartile. aa: amino acid, AAA: aromatic amino acids, AST: aspartate aminotransferase, CRP: C-reactive protein, DPB: diastolic blood pressure, GGT: gamma-glutamyl transpeptidase, glu: glucose, HDL-c: high-density lipoprotein cholesterol, HHRS: high homeostatic resilience score, HMS: high mixed-score, LDL-c: low-density lipoprotein cholesterol, LHRS: low homeostatic resilience score, lip: lipid, LHPS: low health phenotype score, SAA-1: serum amyloid protein-1, SBP: systolic blood pressure, TAG: triacylglycerol, TCH: total cholesterol.


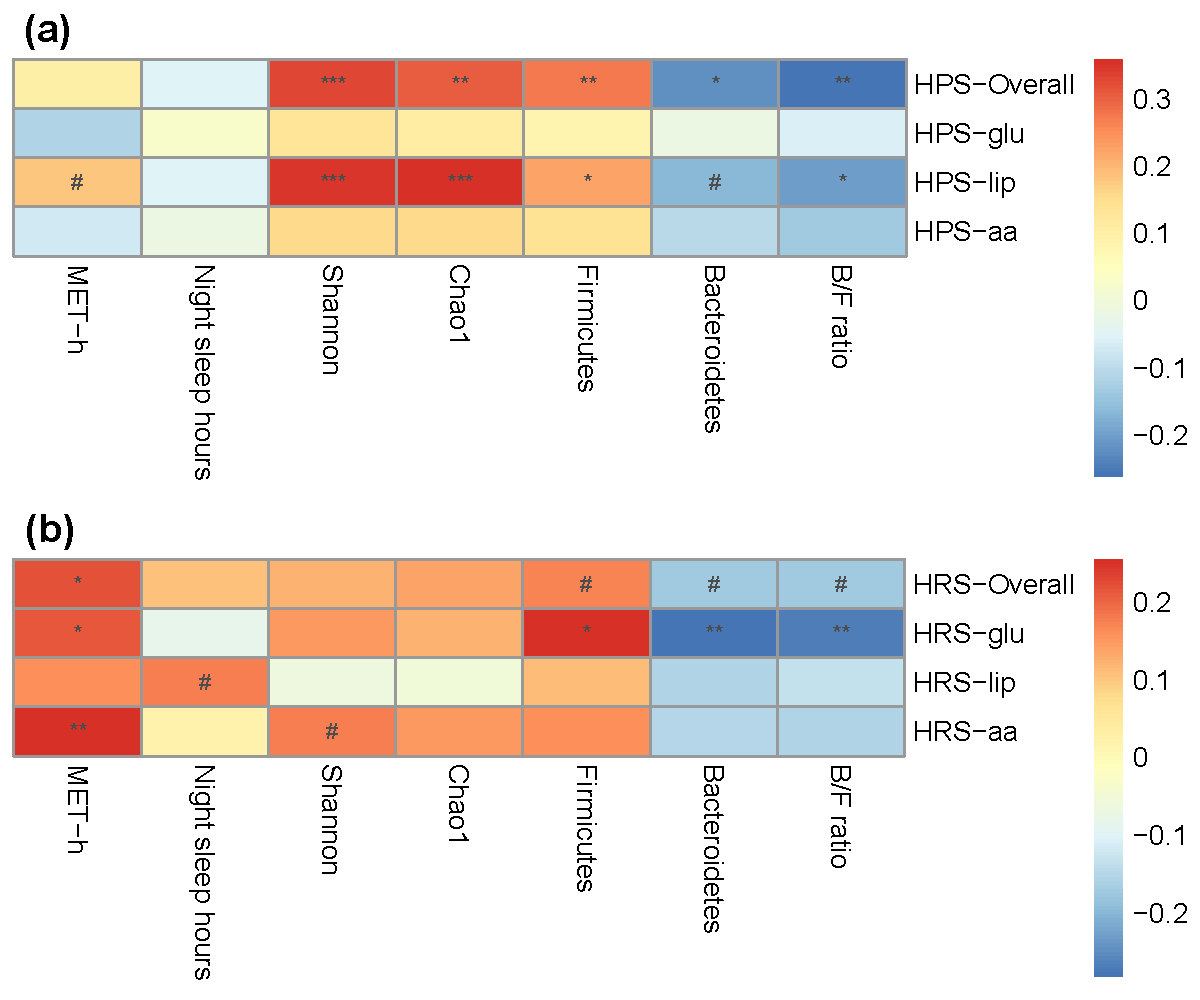


**Fig. S4 Partial Spearman correlations between HPS and lifestyle and microbiome features, adjusted for HRS (a), between HRS and lifestyle and microbiome features, adjusted for HPS (b), (n = 111 for MET-h and Night sleep hours, and n = 107 for microbiota features).** aa: amino acid, B/F ratio: Bacteroidetes/Firmicutes ratio, glu: glucose, lip: lipid, MET-h: metabolic equivalent task hour.


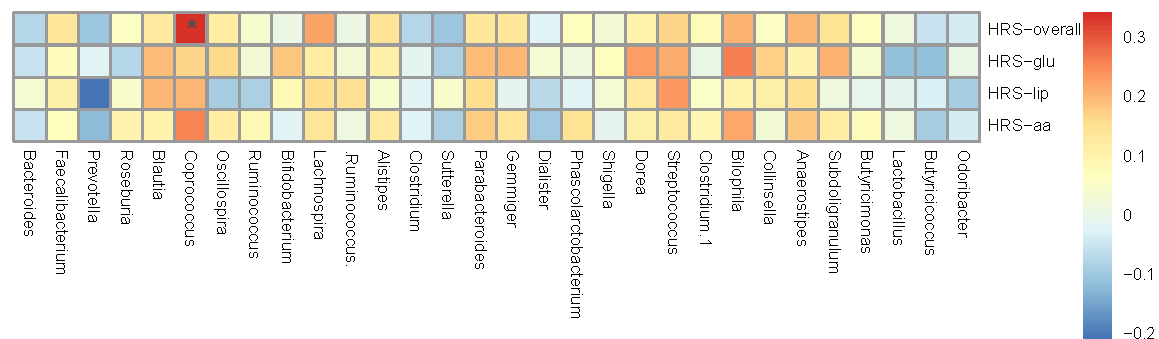


**Fig. S5 Partial Spearman’s correlation coefficients between HRSs and abundances of genera adjusted for age, sex, and BMI (n = 107).** The FDR was computed by the Benjamini–Hochberg method: * < 0.05. aa: amino acid, glu: glucose, HRS: homeostatic resilience score, lip: lipid, Spearman’s *r*: Spearman’s correlation coefficient.


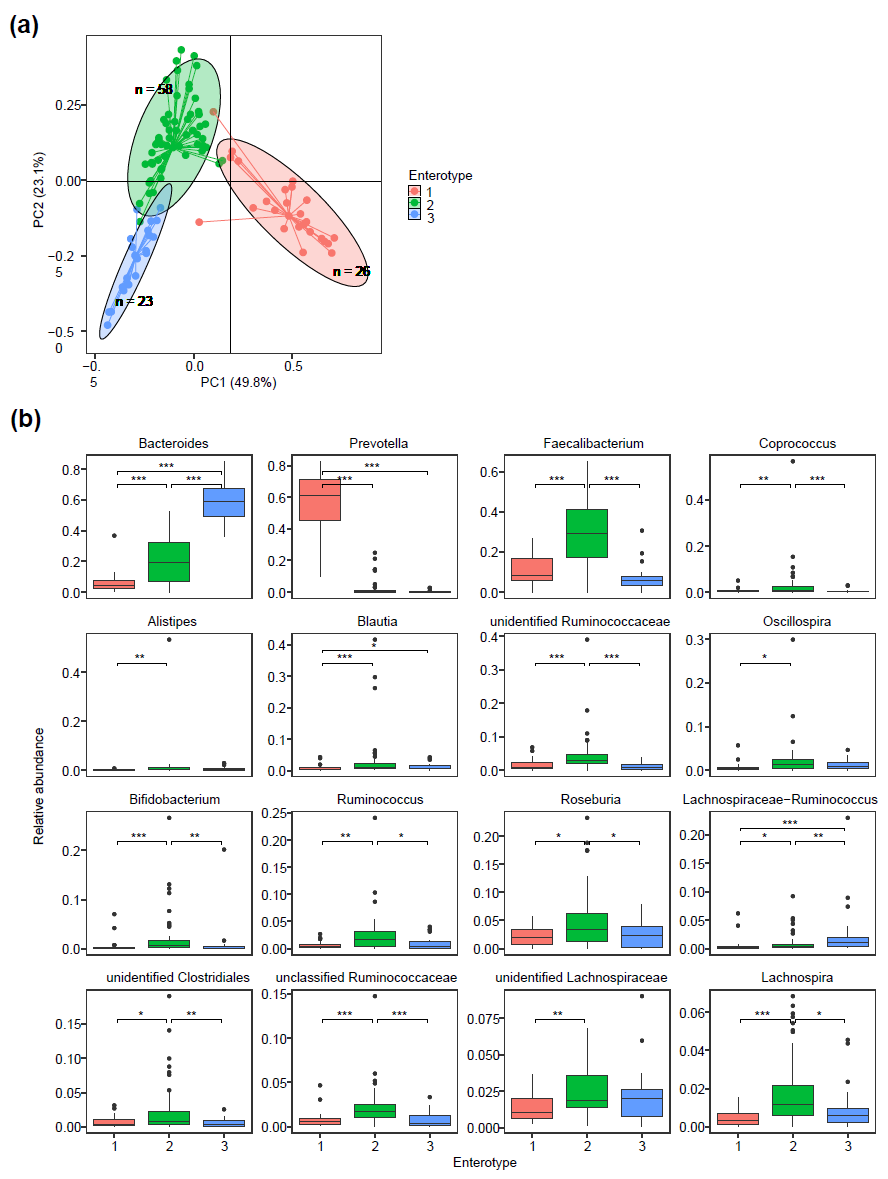


**Fig. S6 Clusters of the three enterotypes at the genus level as represented by the Jensen‒Shannon distance (n = 107).** (**a)** Clusters of the three enterotypes according to the first two principal components. (**b)** Relative abundances of genera in three enterotypes are shown in box and whisker plots. Only those genera with significant differences are presented. Differences in genera among the three enterotypes were tested using the Kruskal‒Wallis test followed by Dunn's post hoc test. The FDR was computed using the Benjamini–Hochberg method: * *P* < 0.05, ** < 0.01, *** < 0.001. PC: principal component.


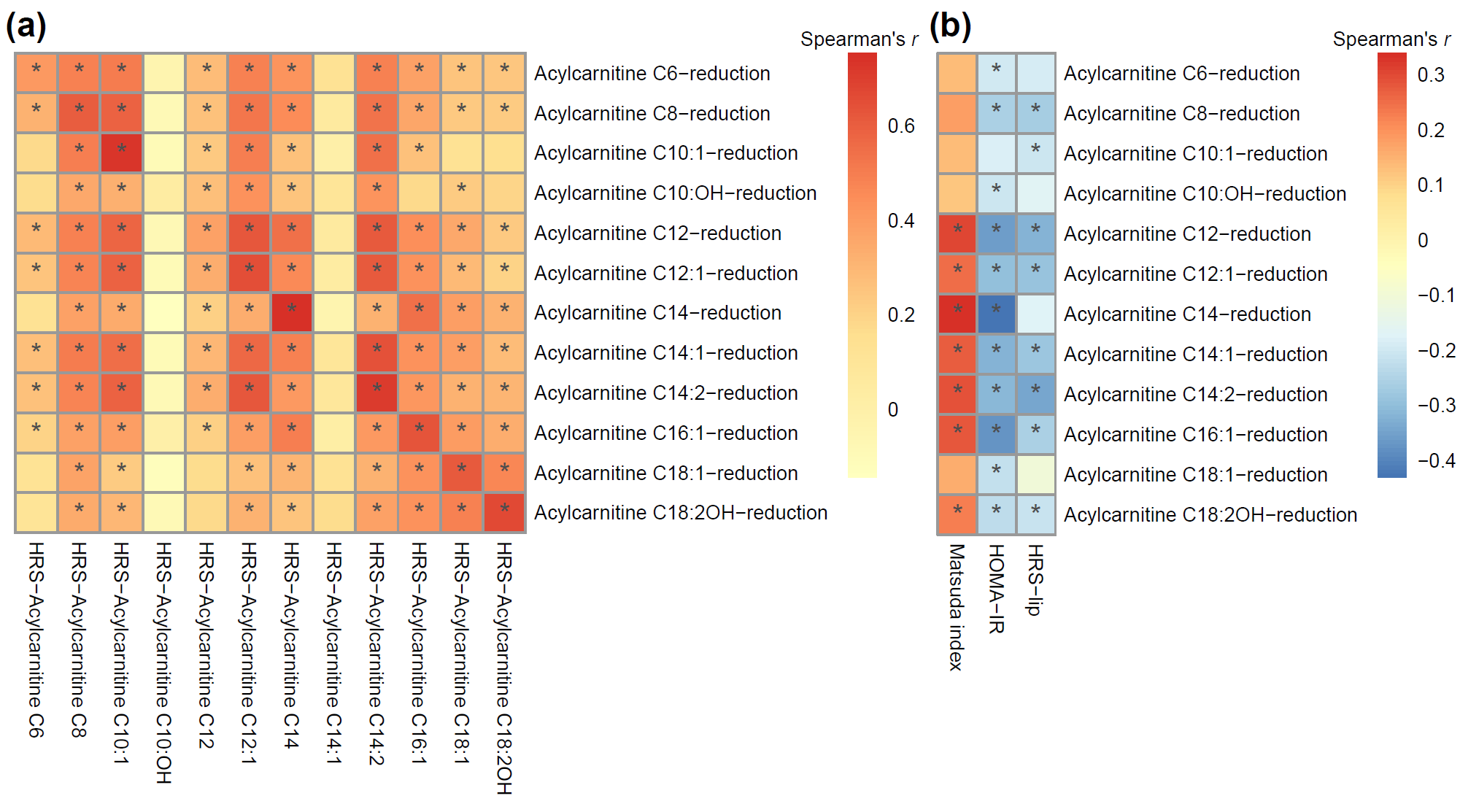


**Fig. S7 Spearman correlation coefficients between decreased acylcarnitine levels post-MMTT and conventional metabolic traits: (a) the HRSs of acylcarnitines with decreased acylcarnitines and (b) decreased acylcarnitines with Matsuda index, HOMA-IR, and HRS-lip values (n = 111)**. The FDR was computed using the Benjamini–Hochberg method: * < 0.05. Acylcarnitine reductions were calculated by subtracting the nadir levels (at 180 minutes post-MMTT) from the fasting levels. HRS: homeostatic resilience score, lip: lipid, Spearman’s *r:* Spearman’s correlation coefficient.


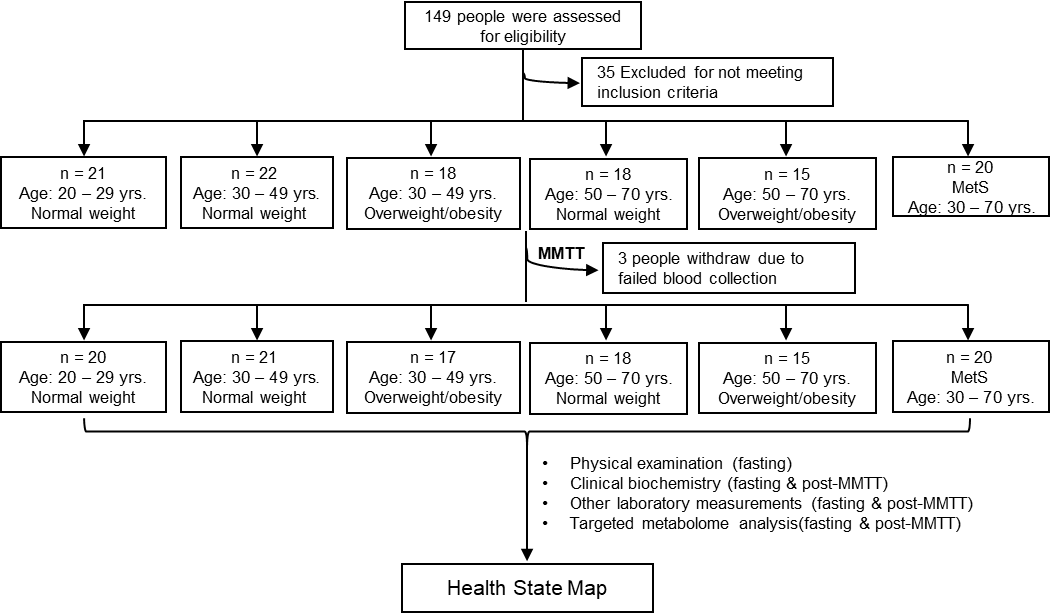


**Fig. S8 Trial flow diagram** The criteria for Chinese individuals were as follows: normal weight, 18.5 kg/m^2^ ≤ BMI < 24 kg/m^2^ and overweight/obese, ≥ 24 kg/m^2^. BMI: body mass index, MMTT: mixed macronutrient tolerance test, MetS: metabolic syndrome, MMTT: mixed macronutrient tolerance test.


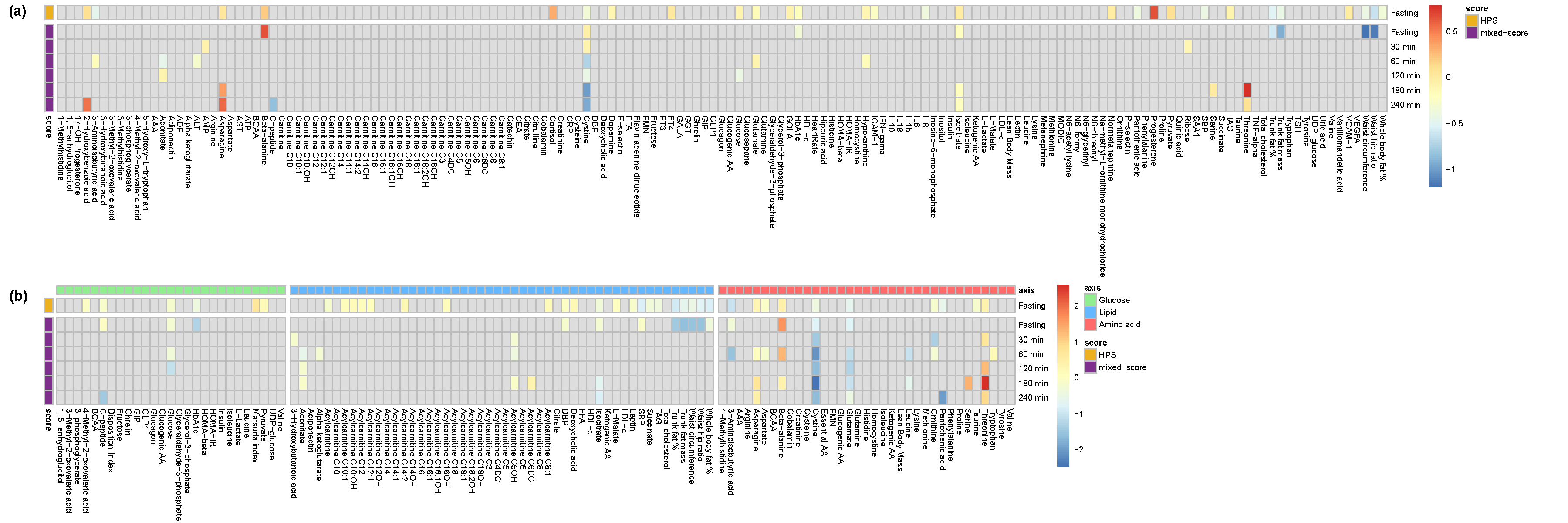


**Fig. S9 Coefficients of features in the HPSs and the mixed-scores on the (a) overall and (b) three axes.** AA: amino acid, AAA: aromatic amino acid, ADP: adenosine diphosphate, ALT: alanine aminotransferase, AMP: adenosine monophosphate, AST: aspartate aminotransferase, ATP: adenosine triphosphate, BCAA: branch chain amino acid, CEA: N7-carboxyethylarginine, CRP: C-reactive protein, DBP: diastolic blood pressure, FFA: free fatty acid, FT3: free triiodothyronine, FT4: free thyroxine, GALA: glycolic acid lysine amide, GGT: gamma-glutamyl transpeptidase, GIP: gastric inhibitory polypeptide, GLP-1: glucagon-like peptide-1, GOLA: glyoxal lysine amide, HDL-c: high-density lipoprotein cholesterol, HPS: Health Phenotype Score, HPS: Health Phenotype Score, ICAM-1: intercellular cell adhesion molecule-1, IFN-g: interferon gamma, IL: interleukin, LDL-c: low-density lipoprotein cholesterol, MODIC: methylglyoxal imidazolinone, SAA-1: serum amyloid protein-1, SBP: systolic blood pressure, TAG: triacylglycerol, TNF-a: tumour necrosis factor-α, TSH: thyroid-stimulating hormone, VCAM-1: vascular cell adhesion molecule-1, VEGFA: vascular endothelial growth factor receptor.


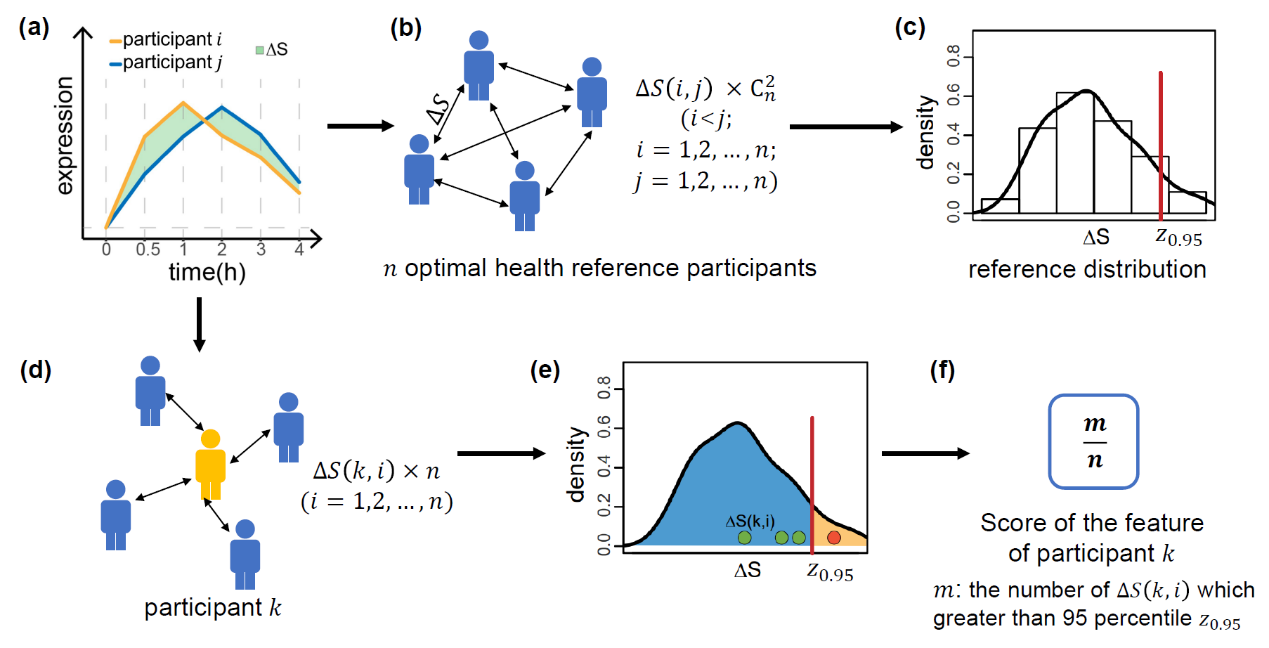


**Fig. S10 Flow chart of the** **area between response curves (ABRC) method. (a)** For each feature *g* of one participant, a response curve was generated to describe the fluctuation of feature *g* during the standard oral mixed macronutrient tolerance test (MMTT) by using changed levels of the feature measured at 6 time points. The area (∆S) between the response curves of participants *i* and *j* characterized their differences in homeostatic resilience. **(b)** It was assumed that the predefined optimal health reference group had optimal homeostatic resilience; thus, we calculated the ∆S between every pair of *n* participants in the optimal health reference group to form a reference ∆S vector containing $C_{n}^{2}$ elements. The reference ∆S represents the difference between the participants with the optimal homeostatic resilience. **(c)** The 0.95 quantile ($z_{0.95}$) was calculated for the reference distribution and is shown here as a red line. The difference between the two curves was significant when ∆S was > $z_{0.95}$. **(d)** The *n* ∆S between the response curve of participant *k* and *n* participants in the optimal health reference group was calculated to represent the differences in homeostatic resilience between participant *k* and every reference participant. **(e)** The *n* ∆S of participant *k* was compared with the $z_{0.95}$ of the reference distribution. ∆S > $z_{0.95}$, as represented by the red dot, meant that the response curve between participant *k* and the reference participant significantly differed. **(f)** The resilience score of feature *g* of participant *k* was computed as the proportion of patients with ∆S values ≤ $z_{0.95}$. The proportion was calculated by $(n-m)/n$, where $m$ is the number of ∆S values > $z_{0.95}$. A larger proportion indicated a smaller difference between participant *k* and the reference participants.

1. Zhou B; Prospective study for cut-off points of body mass index in Chinese adults. *Zhonghua Liu Xing Bing Xue Za Zhi* 2002;**23**:431-4.

2. van den Broek TJ, Bakker GCM, Rubingh CM, et al.; Ranges of phenotypic flexibility in healthy subjects. *Genes Nutr* 2017;**12**:32.

3. Ye X, Yu Z, Li H, et al.; Distributions of C-reactive protein and its association with metabolic syndrome in middle-aged and older Chinese people. *J Am Coll Cardiol* 2007;**49**:1798-805.

4. Sjostrom M, Ainsworth BE, Bauman A, et al.; Guidelines for data processing analysis of the International Physical Activity Questionnaire (IPAQ) - Short and long forms. 2005.

5. Matthews DR, Hosker JP, Rudenski AS, et al.; Homeostasis model assessment: insulin resistance and beta-cell function from fasting plasma glucose and insulin concentrations in man. *Diabetologia* 1985;**28**:412-9.

6. Matsuda M, DeFronzo RA; Insulin sensitivity indices obtained from oral glucose tolerance testing: comparison with the euglycemic insulin clamp. *Diabetes Care* 1999;**22**:1462-70.

7. Ma YC, Zuo L, Chen JH, et al.; Modified glomerular filtration rate estimating equation for Chinese patients with chronic kidney disease. *J Am Soc Nephrol* 2006;**17**:2937-44.

8. Chen J, Spracklen CN, Marenne G, et al.; The trans-ancestral genomic architecture of glycemic traits. *Nat Genet* 2021;**53**:840-860.

9. Wojczynski MK, Parnell LD, Pollin TI, et al.; Genome-wide association study of triglyceride response to a high-fat meal among participants of the NHLBI Genetics of Lipid Lowering Drugs and Diet Network (GOLDN). *Metabolism* 2015;**64**:1359-71.

10. Prokopenko I, Poon W, Magi R, et al.; A central role for GRB10 in regulation of islet function in man. *PLoS Genet* 2014;**10**:e1004235.

11. Bolyen E, Rideout JR, Dillon MR, et al.; Reproducible, interactive, scalable and extensible microbiome data science using QIIME 2. *Nat Biotechnol* 2019;**37**:852-857.

12. MARTIN M; Cutadapt removes adapter sequences from high-throughput sequencing reads. *EMBnet.journal* 2011;**17**.

13. Grundy SM, Cleeman JI, Daniels SR, et al.; Diagnosis and management of the metabolic syndrome: an American Heart Association/National Heart, Lung, and Blood Institute Scientific Statement. *Circulation* 2005;**112**:2735-52.

14. Chen L, Liu R, Liu ZP, et al.; Detecting early-warning signals for sudden deterioration of complex diseases by dynamical network biomarkers. *Sci Rep* 2012;**2**:342.

15. Shi J, Li T, Chen L, et al.; Quantifying pluripotency landscape of cell differentiation from scRNA-seq data by continuous birth-death process. *PLoS Comput Biol* 2019;**15**:e1007488.

16. Shi J, Aihara K, Li T, et al.; Energy landscape decomposition for cell differentiation with proliferation effect. *Natl Sci Rev* 2022;**9**:nwac116.

17. Hoevenaars FPM, Esser D, Schutte S, et al.; Whole Grain Wheat Consumption Affects Postprandial Inflammatory Response in a Randomized Controlled Trial in Overweight and Obese Adults with Mild Hypercholesterolemia in the Graandioos Study. *J Nutr* 2019;**149**:2133-2144.

18. Chinese Guidelines for Primary Prevention of cardiovascular diseases. *Practical Journal of Cardiac Cerebral Pneumal and Vascular Disease* 2021;**29**:44+64.

19. Endocrinology CSo; Guidelines for diagnosis and treatment of adult hypothyroidism. *Chin J Endocrinol Metab* 2017:167-180.

20. Levey AS, de Jong PE, Coresh J, et al.; The definition, classification, and prognosis of chronic kidney disease: a KDIGO Controversies Conference report. *Kidney Int* 2011;**80**:17-28.
